# Interpersonal vs. supportive group psychotherapy for depression attributed to work stress: study protocol of the multicentre, cluster-randomised, controlled IPT-Work trial

**DOI:** 10.1186/s12888-025-06594-w

**Published:** 2025-02-19

**Authors:** Elisabeth Schramm, Moritz Elsaesser, Nadine Zehender, Andreas Reif, Claas Lahmann, Manon Feuchtinger, Michael Deuschle, Kai Kahl, Andreas Hillert, Nicola Thiel, Hannah Piosczyk, Simon Mack, Johannes Bausch, Erika Graf

**Affiliations:** 1 Department of Psychiatry and Psychotherapy, Medical Center – University of Freiburg, Faculty of Medicine, University of Freiburg, Freiburg, Germany; 2 Department of Psychiatry, Psychosomatic Medicine and Psychotherapy, University Hospital Frankfurt-Goethe University, Frankfurt, Germany; 3 Department for Psychosomatic Medicine and Psychotherapy, Medical Center, University of Freiburg, Faculty of Medicine, University of Freiburg, Freiburg, Germany; 4 Central Institute of Mental Health, Medical Faculty Mannheim/Heidelberg University, Mannheim, Germany; 5 Department of Psychiatry, Social Psychiatry and Psychotherapy, Hannover Medical School, Hannover, Germany; 6 Schön Klinik Roseneck, Prien Am Chiemsee, Germany; 7 Hochschule Fresenius Heidelberg, Heidelberg, Germany; 8 Clinical Trials Unit, Medical Center, Faculty of Medicine, University of Freiburg, Freiburg, Germany; 9 Institute of Medical Biometry and Statistics, Medical Center – University of Freiburg, Faculty of Medicine, University of Freiburg, Freiburg, Germany

**Keywords:** Work stress, Depression, Work-directed interventions, Group treatment, Work outcomes, Interpersonal psychotherapy, IPT-work, Supportive psychotherapy, Psychotherapy, Randomised controlled trial

## Abstract

**Background:**

Depression associated with occupational stress is highly prevalent, causing high rates of sick leave and thus posing significant societal and economic burden. Meta-analyses of the few studies on psychological and work-focused interventions for common mental disorders including depression report small effects on depressive symptomatology and occupational outcomes. There is an urgent need for more controlled studies on work-directed interventions assessing work outcomes.

**Methods:**

This is an interventional, multicentre, active-controlled, cluster-randomised, observer-blinded clinical trial with two parallel groups conducted in 6 clinical centres throughout Germany over the course of 3 years. A sample of 144 outpatients with work stress related depression will be cluster-randomised to either a specific interpersonal group intervention for depression and work stress (IPT-Work) or a nonspecific supportive group psychotherapy (SP). Each group consists of 10 sessions over 8 weeks of 90 min duration with 4–6 participants. Patients will be assessed at baseline, post-treatment and at 3 months follow-up. The primary endpoint is the relative change in HRSD-24 score from baseline to follow-up 3 months after end of treatment. Secondary outcome measures include the Occupational Depression Inventory (ODI), the Work Ability Index (WAI), the Return to Work Attitude (RTW-SE), the Effort-Reward-Imbalance (ERI), the Job Content Questionnaire 2 (JCQ2), and the Connor-Davidson Resilience Scale (CD-RISC). In addition, Quality of Life (WHOQOL-BREF) and days of sick leave throughout the study period will be assessed.

Effects of treatment will be analysed with a linear mixed model for repeated measures including randomised arm, time point and their interaction as well as HRSD-24 baseline scores and their interaction with time point as fixed effects.

**Discussion:**

Results will provide a comparison of a nonwork-directed psychological intervention and a specific, work-directed approach with respect to symptom improvement and increase in work ability. The aim is to improve quality of mental health care for depressed employees to facilitate recovery, improve work ability, and reduce the risk of long-term occupational incapacity. Ultimately, findings will inform the practice of the efficiency of using psychological group treatment in depressed individuals with work stress.

**Trial registration:**

German Clinical Trials Register (DRKS00035259); prospectively registered on 15th January 2025.

**Supplementary Information:**

The online version contains supplementary material available at 10.1186/s12888-025-06594-w.

## Background

Around 15% of people at work were estimated to have a mental disorder [1] with depression being most prevalent while affecting every 10th female and every 20th male worker [2]. The 12-month prevalence of major depressive disorder (MDD) among employees has been reported at 7.6% [3]. Depressive disorders have a major impact on social and occupational functioning [4] and are increasingly recognised as a significant mental health problem contributing to major productivity loss and economic burden to organisations [1, 5]. MDD are among the leading causes of sick leave and long-term work incapacity in most modern countries. Administrative data from national health statistics document a fourfold increase in days of sick leave due to mental disorders, particularly depression, between 1997 and 2023 [6]. In a scoping review, the majority of the 125 included studies report significant associations between work-related stress and depression [7]. At the same time, positive effects of “good” work on mental health and the important role work can play in enhancing mental well-being and facilitating recovery from a mental illness are described [8].

### Specific work-directed interventions

There is an urgent need to evaluate innovative treatments for work-related depression by adapting existing effective interventions to focus on the work context and to include work-related outcomes [9]. The best investigated predictors for depression in the context of work stress are psychosocial in nature and include high job demands in connection with low decision latitude (demand-control-imbalance), lack of gratification (effort-reward-imbalance), low social support, interpersonal conflicts, role stress, and organisational injustice [8]. For the specific treatment of workplace depression, a novel focus “work stress” of Interpersonal Psychotherapy (IPT-Work) was conceptualised addressing those psychosocial stressors as work usually takes place in an interpersonal context. IPT is a first line treatment for depression [10] whose effectiveness has been demonstrated for the four standard foci “interpersonal disputes”, “role transitions”, “social deficits”, and “grief”. There is preliminary evidence [11, 12] that the additional focus of “work-stress” including mindfulness techniques is an appropriate fit for the therapy of occupational problems associated with the depressive episode.

Other recent findings suggest that workplace directed interventions facilitate the recovery of employees diagnosed with MDD and produce beneficial effects on occupational outcomes, particularly when combined with clinical interventions (e.g. antidepressant medication) [9, 13]. However, the small number of controlled studies on the effects of specific psychotherapy on work-related outcomes in depression makes it difficult to draw final conclusions.

### Pilot study

In a pilot study, we evaluated the feasibility and generated first data on the effectiveness of IPT adapted for a group setting to focus on the work context (IPT-Work) [11]. In total, 28 outpatients (22 women; M = 49.8 years old) with MDD related to work stress were randomised to 8 weekly group sessions of IPT-Work or to treatment as usual (TAU; guideline oriented treatment; might include pharmacotherapy and/or psychotherapy). Primary endpoint was the Hamilton Rating Scale for Depression (HRSD-24) [14] score. Key secondary endpoints were, among others, the Beck Depression Inventory (BDI-II) [15], the Work Ability Index (WAI) [16]), the Return to Work Attitude (RTW-SE) [17], and the Effort-Reward-Imbalance (ERI) [18]. In addition, we evaluated the participants’ overall satisfaction with the IPT-Work programme. A follow-up assessment was conducted 3 months after end of acute treatment. IPT-Work was significantly more effective than TAU in reducing clinician-assessed depressive symptoms at follow-up and self-assessed depression at both endpoints. Furthermore, IPT-Work was superior in improving the work-ability, the return-to-work attitude, and the effort-reward-ratio at work. The vast majority (89 percent) of participants in the W-IPT condition were “very satisfied” with the programme, although wishing for a greater number of sessions (75 percent). No dropouts were observed in both groups. In summary, a work-focused IPT programme for the treatment of depression associated to work stress was feasible and highly acceptable. IPT-Work turned out to be more effective than standard treatment in reducing depression and problems at work.

### Changes in the multicentre trial

For the confirmatory trial presented here, we discarded TAU as a control condition and will use Supportive Psychotherapy (SP) [19] instead since a TAU group might be very heterogeneous, less intense and is susceptible to bias. It could not be distinguished if the observed effect is caused by the treatment itself or simply by the different treatment intensities. By using SP as a nonspecific, non-work-directed, but effective intervention [20], treatment intensity is equal, and in both conditions no other concurrent psychotherapy outside the study participation is allowed.

SP will be applied in the same format (group sessions), frequency and duration as the IPT-Work condition in order to implement a homogeneous and comparable control group. Since SP is based on exclusively common therapy effects (common factors approach) and IPT-Work elicits common as well as specific therapy effects, using SP as control group also enables an estimate of the specific effects of IPT-Work beyond the common therapy effects.

Our primary hypothesis is that IPT with a specific focus on work stress (IPT-Work) is more effective in reducing depressive symptoms compared to SP 3 months after end of treatment. Furthermore, we predict that IPT-Work will as well increase work ability.

## Methods

### Study design

This is an interventional, multicentre, national, cluster-randomised, active-controlled, observer-blinded clinical trial with two parallel groups conducted in six clinical centres throughout Germany. A sample of 144 patients with depression and work stress (see exclusion/inclusion criteria below) will be randomised to either a manual-based interpersonal group intervention or a manual-based nonspecific supportive group intervention in groups of 4 to 6 outpatients. Each group will consist of 10 sessions over 8 weeks (twice weekly in the first 2 weeks and weekly thereafter) of 90 min duration. Patients will be assessed at baseline, post-treatment and at 3 months follow-up. The primary endpoint is the relative change in HRSD-24 score from baseline to follow-up 3 months after end of treatment (secondary outcomes see below). The trial was prospectively registered at the German Clinical Trials Register (DRKS00035259) on 15th January 2025.

### Inclusion and exclusion criteria

All participants need a primary diagnosis of Major Depression (single-episode or recurrent) according to the Structured Clinical Interview for DSM-5 (SCID-5-CV) [21], a score of ≥ 17 on the 24-item version of the Hamilton Rating Scale for Depression (HRSD-24) [14] and a total score of at least 15 on the Occupational Depression Inventory (ODI) [22]. Additionally, all participants need at least 7 days of sick leave related to depressive or burn-out complaints during the last 12 months and must have a regular work activity that is expected to continue for at least 6 months at the time of randomisation. Sufficient German language skills, outpatient status, an age of 18-years or older and patient’s written informed consent are mandatory for study inclusion.

Participants will be excluded if any of the following criteria apply to them: Acute risk of suicide; history of psychotic symptoms, bipolar disorder, or organic brain disorders; a primary diagnosis of another SCID-5-CV disorder; concurrent diagnosis of substance dependency; antisocial, schizotypal, or borderline personality disorder (SCID-5-PD) [21]; other ongoing psychotherapy; antidepressive pharmacotherapy (if not stable for at least the last 4 weeks before randomisation); serious medical condition or time restrictions interfering with participation in regular sessions; current sick leave > 4 weeks; applying for rehabilitation or early retirement; patient without legal capacity who is unable to understand the nature, significance and consequences of the study; simultaneous participation in other studies which could interfere with this study and/or participation before the end of a required restriction period; persons who are in a disciplinary employment relationship with a member of the study team.

### Sample size calculation

**Fig. 1:**
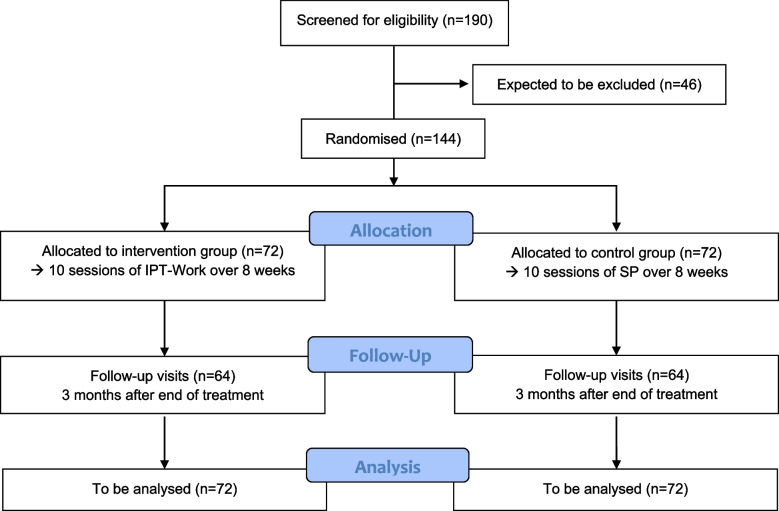
Trial flowchart

In the pilot trial [11], mean ± SD relative changes (decreases) from baseline to follow-up of HRSD-24 were 62.2% ± 28.0% and 22.6% ± 71.6% for IPT-Work and TAU. On the basis of a two-group t-test at two-sided significance level of 5%, a sample size of 62 observations per arm yields a power of 80% to detect a difference if the HRSD-24 mean relative changes from baseline at follow-up are assumed to differ by 28.0% points with a common SD of 55.0% (nQuery Version 9.2.0.0). The common SD is estimated to lie in between SDs seen in the pilot trial for IPT-Work and TAU, since IPT-Work and SP have similar treatment intensity. The means difference is considered both minimum clinically relevant [23] and realistic since IPT is generally more effective than SP [24] and IPT-Work is specifically tailored to depression in a work context. This calculation is conservative given that evaluation in a linear model adjusting for baseline measurement will further increase the power. Due to IPT-Work and SP group sizes (clusters) of only 4–6 participants and the fact that the interventions are directed at patients, the design effect induced by intracluster correlation is conservatively estimated as 1.0438, yielding an increase to 62 × 1.04≈64 observations per arm. Given an anticipated drop-out rate at follow-up of 10%, 128/0.9≈144 patients should be randomised. Therefore, about 190 patients will be assessed for eligibility (Fig. 1).

### Randomisation

Groups of 4–6 eligible patients at a time, recruited by blinded raters, will be cluster-randomised to the IPT-Work or SP condition. Central 1:1 allocation of patient clusters, stratified by study site and using variable, non-disclosed block lengths, will be performed according to a computer-generated sequence at the Clinical Trials Unit Freiburg upon request by the study psychotherapists. Effective concealment is ensured through restricted access to randomisation data, strict confidentiality duties for site personnel, and blinding of raters to treatment allocations of ongoing or previous therapy groups.

### Primary and secondary endpoints

The primary outcome is the relative change in the clinician-rated 24-item Hamilton Rating Scale for Depression (HRSD-24) score from baseline to 3 months after end of treatment, calculated as (100–(100xpost/pre)%). The HRSD-24 is the most frequently used measure in depression research and covers the most domains relevant to patients with depression in comparison to other outcome measure [25]. Recent investigations show that the HRSD is a valid and sensitive clinimetric index when conducted by trained raters using a structured form [26]. Higher values of the HRSD-24 correspond to greater severity of depressive symptoms. There are no subscales. Further secondary endpoints are listed in Table 1.

**Table 1:** Primary and further secondary endpoints and corresponding measures

**Primary Endpoint**	**Measures**
Clinician-rated relative change in depression severity at post-treatment (secondary endpoint) and 3 months follow-up (primary endpoint).	*24-item Hamilton Rating Scale for Depression* (HRSD-24) [14] rated by trained and blinded clinicians. Higher scores correspond to greater severity.
**Secondary Endpoints**	**Measures**
Clinician-rated response and remission rate at post-treatment and follow-up	Response is defined as a reduction in the HRSD-24 score by at least 50% from baseline. Remission is defined a priori as an HRSD-24 score of < 9.
Self-rated work-attributed depressive symptoms at post-treatment and follow-up, absolute change from baseline	The *Occupational Depression Inventory* (ODI) [22] is a self-rated 10-item questionnaire to assess work-attributed depressive symptoms. Higher scores correspond to greater severity.
Self-rated depression severity at post-treatment and follow-up	The *Beck Depression Inventory-II* (BDI-II) [15] is a self-rated 21-item questionnaire to assess depression severity. Higher scores correspond to greater severity
Self-rated work ability at post-treatment and follow-up, absolute change from baseline	The *Work Ability Index* (WAI) [16] is a self-rated 7-domain questionnaire to assess work ability. Higher scores correspond to greater work ability.
Self-rated return-to-work self-efficacy at post-treatment and follow-up, absolute change from baseline	The *Return to Work Attitude* (RTW-SE) [17] is a self-rated 11-item questionnaire to assess return-to-work self-efficacy for employees with mental health problems. Higher scores correspond to higher self-rated return-to-work self-efficacy.
Self-rated imbalances between work-related efforts and rewards at post-treatment and follow-up	The *Effort-Reward-Imbalance* (ERI) [18] is a self-rated 22-item questionnaire to assess imbalances between work-related efforts and rewards. Higher scores correspond to a higher effort-reward-imbalance. There are three subscales assessing effort, reward and over-commitment.
Self-rated JCQ2 at post-treatment and follow-up, absolute change from baseline	The *Job Content Questionnaire 2* (JCQ2) [27] is a self-rated questionnaire designed to measure the “content” of a respondent’s work tasks covering critical workplace issues that are often overlooked (such as employee health risks, chronic disease, sickness-absence, disability, and job satisfaction). It is used to measure the high-demand/low-control/low-support model of job strain development and predicts stress-related risk and active–passive behavioral correlates of jobs according to the demand/control model. Higher scores correspond to higher job strain.
Self-rated quality of life at post-treatment and follow-up, absolute change from baseline	The *WHO Quality of Life Instrument* (WHOQOL-BREF) [28] is a self-rated 26-item questionnaire divided to measure quality of life in four domains (physical health, psychological health, social relationships and the environment). Higher scores correspond to higher domain-specific quality of life.
Self-rated resilience at post-treatment and follow-up, absolute change from baseline	The Connor-Davidson Resilience Scale (CD-RISC) [29] is a self-rated 10-item questionnaire to assess resilience. Higher scores correspond to greater resilience.

In addition, exploratory endpoints include the number of self-reported days of sick leave throughout the study period. Safety is assessed in terms of rates of adverse events and serious adverse events.

In the primary and secondary efficacy estimands, treatments will be compared as randomised, regardless of treatment interruptions, discontinuation or prohibited concomitant treatments. These are the only relevant intercurrent events, and they will be addressed by the treatment policy strategy [30]. The population summary measure to compare IPT-Work and SP is the difference of means for change from baseline endpoints and the odds ratio for response and remission.

### Trial visits

Patients will be assessed at baseline, post-treatment and 3 months follow-up. The detailed overview on the frequency and scope of the trial visits is depicted in Table 2.

**Table 2:** Frequency and scope of trial visits

Trial Period	Baseline		Treatment						Post-treatment	Follow-up/ End of Trial
	**Screening 14 days**	**Randomisation**	**8 weeks**						**13 weeks**	
**Visits**	V0	V1	V2	V3	V4	V5	V6-V10	V11	V12	V13
**Week**		W0	W1		W2		W3-7	W8	W9	W21
**Time Window**	day -14 — day 0	day 0	± 4 days	± 4 days	± 7 days	± 7 days	± 7 days	± 7 days	± 7 days	± 7 days
Informed Consent	x									
Inclusion/Exclusion Criteria	x									
Demographics / Medical History	x									
Work Context	x								x	x
Structured Clinical Interview for DSM-5 (SCID-5)^b^	x									
Hamilton Rating Scale for Depression (HRSD-24)	x								x	x
Occupational Depression Inventory (ODI)^a^	x								x	x
Days of sick leave^a^	x								x	x
Confirmation of SCID-5, HRSD-24, ODI (if older than 14 days: re-assessment)		x								
Beck Depression Inventory II (BDI-II)^a^	x								x	x
Work Ability Index (WAI)^a^	x								x	x
Return to Work Attitude (RTW-SE)^a^	x								x	x
Effort-Reward-Imbalance (ERI) at work^a^	x								x	x
Job Content Questionnaire 2 (JCQ2)^a^	x								x	x
WHOQOL-BREF (Quality of Life)^a^	x								x	x
Connor-Davidson Resilience Scale (CD-RISC)^a^	x								x	x
Patient treatment preference^a^	x								x	
Randomisation^b^		x								
Individual preliminary talk^b^			x							
Treatment IPT-Work vs SP sessions^b^			x	x	x	x	x	x		
Therapeutic element checklist (Stundenbogen)^b^			x	x	x	x	x	x		
Change of medication/therapy^b^									x	x
Adverse Events / Serious Adverse Events^b^			x	x	x	x	x	x	x	x

*Vx* visit x, *Wx* week x, *DSM-5* Diagnostic and Statistical Manual of Mental Disorders, Fifth Edition

^a^Self-rated by patient

^b^after informed consent

### Treatment

Before the start of the group treatment, in both study arms one individual session will be conducted in which the history of the illness in the work context is explored, individual goals are set, and the necessity of involving a social worker in the treatment (to target specific social law related workplace issues) is determined.

#### Experimental intervention: IPT-Work

The IPT-Work condition follows a manual and focuses on the work context by adding specific elements to the regular IPT strategies in four modules of.

1. work-life-balance: identifying work-related stress factors, dysbalances, and allostatic overload; psychoeducation on the health stabilizing effects and functions of a positive work role; psychoeducation on the association of work stress, social support and depression; creating a balance between performance values and interpersonal values; teaching mindfulness skills (as a different therapeutic element) to reduce physical and mental tension/stress; establishing social support at the workplace and outside the workplace;
2. demand-control-balance: enhancing communication skills at work (e.g. negotiating modified work tasks or working hours, asking for adequate gratification) in role plays to cope with difficult or stressful work situations; set limits to exaggerated demands (prevent allostatic overload; get a sense of control over the work conditions e.g. organisational injustice); cope with interpersonal conflicts and difficult role transitions at the workplace;
3. effort-reward-balance: identify values at work; set self-exertion and reward in balance; honour your values at work and cope with situations where your values are hurt;
4. work as a “social role”: define your work place with strengths and limitations.

#### Control intervention: Supportive Psychotherapy (SP)

SP is a manual-based [19], nonspecific, nondirective psychotherapeutic intervention found to be effective in the treatment of depression [20, 24]. It resembles supportive clinical management or client-centred counselling and includes psychoeducational elements and other common aspects of psychotherapy, such as reflective listening, empathic responding, motivational support, facilitation of affect, helping the patient to feel understood, instilling hope, and therapeutic optimism. Specific interpersonal, cognitive, behavioural, systemic and psychodynamic interventions are explicitly proscribed.

#### Psychotherapists

Study psychotherapists are in a completed or far advanced stage of psychotherapy training. All therapists will execute both IPT-Work as well as SP groups after thorough training (2-day training in presence in each IPT-Work and SP followed by 3 half-day online booster workshops). To check for adherence and to support the supervision, a ‘Therapeutic Element Checklist’ for IPT-Work (e.g. strategies for addressing work-life-balance, demand-control-balance, effort-reward-balance, and work as a social role) and SP (e.g. reflective listening, facilitation of affect, helping the patient to feel understood) is filled out by the therapist immediately after each group session. Separate supervisors for each treatment arm will review the ‘Therapeutic Element Checklist’ regularly in ongoing supervision. Every second session will be supervised by the responsible supervisors in biweekly video conference meetings.

#### Raters

All raters have at least a B.Sc. in Psychology (or comparable), are trained in a 2-day training to assess study participants based on predefined criteria and measurement tools, and remain blind to the participants’ group assignments. Each of the sites implements procedures to mask a patient treatment assignment through the following: (1) Locating the raters at a separate physical location, and (2) reminding the patients at each visit not to mention anything that might reveal their treatment condition to the independent rater. To ensure adherence and interrater reliability, raters have independently rated videos of HRSD-24 gold standard ratings (primary outcome) during the training and will continue to do so during the duration of the trial.

### Recruitment

Participants will be recruited through outpatient centres and private practices (primary care, psychosomatics, or psychiatry), through firms and institutions (such as clinics, schools, universities), as well as through media. In current practice, respective patients are often not evaluated for depression. Hence, a maximum effort is put to exposure of the targeted patient group to the study sites. To do so, a study website is created to provide information about the study. As the general public often regards psychotherapy with or without medication as the preferred treatment option for depression, we will also provide corresponding information for treating physicians.

### Adverse and serious adverse events

For risk assessment and to monitor safety of randomised patients, adverse events (AEs) were defined ahead of the study as any untoward medical occurrence, and criteria for classifying serious adverse events (SAEs) were specified (see supplement for further descriptions). AEs and SAEs will be recorded for each patient from randomisation until end of study (follow-up) at every assessment or therapy session. In case an adverse event meets the seriousness criteria, a SAE-Reporting-Form has to be filled out, and the coordinating investigator and the principal investigator at the site will be notified.

### Data Safety Monitoring Board (DSMB)

An independent DSMB of three independent experts in the field has been established. The function of the DSMB is to monitor the course of the trial and if necessary to give a recommendation to the coordinating investigator for continuation, modification or discontinuation of the trial. The underlying principles for the DSMB are ethical and safety aspects for the patients. It is the task of the DSMB to examine, whether the conduct of the trial is still ethically justifiable, whether safety of the patients is ensured, and whether the process of the trial is acceptable. For this the DSMB will be informed about the patient recruitment, the adherence to the protocol, and the observed adverse events.

### Data management and monitoring

Study data will be entered in pseudonymised form in a study database (eCRF) by authorised and trained members of the study team. The data management will be performed with REDCap V.9, a fully web based remote data entry system based on web forms, which is developed and maintained by the REDCap Consortium (https://projectredcap.org/about/consortium/). This system uses built-in security features to prevent unauthorised access to patient data, including an encrypted transport protocol for data transmission from the participating sites to the study database. An audit trail provides a history of the data entered, changed or deleted, indicating the processor and date. Monitoring is performed by the CTU Freiburg. Risk-based monitoring will be done according to ICH-GCP E6 (R2) and standard operating procedures to ensure patient’s safety and integrity of clinical trial data.

### Discontinuation criteria

The coordinating investigator supported by the DSMB is under obligation to monitor the progress of the study with regard to safety-relevant developments and initiate the premature termination of a treatment arm or the entire study if necessary. A study site or the entire study must be terminated prematurely if the benefit-to-risk ratio for the patients changes markedly, the coordinating investigator or the DSMB considers that the termination of the study is necessary, indications arise that the study patients’ safety is no longer guaranteed, the questions addressed in the study can be clearly answered on the basis of results of another study on the same subjects, or an insufficient recruitment rate makes a successful conclusion of the study unrealisable or no longer feasible. If the study is prematurely terminated or suspended for any reason, the coordinating investigator will promptly inform the concerned ethics committees and ensure appropriate therapy and follow-up for the already randomised patients.

### Patient involvement

The self-help organisation “Deutsche DepressionsLiga e.V.” will coordinate the formation of a steady advisory board of patient representatives. Members of this advisory board were involved in planning and designing the trial, reviewed the study protocol and will be informed about the process of the study and asked to provide input on all relevant issues. The involvement of focus groups with patient representatives is planned. The Deutsche DepressionsLiga will also support in disseminating the project results.

### Statistical analysis

In the primary analysis, all randomised patients will be analysed as belonging to their randomised arm, regardless of any protocol deviations. The effects of SP and IPT-Work on the difference of means [IPT-Work minus SP] of the relative change in HRSD-24 score from baseline to follow-up will be estimated and tested in a linear mixed model for repeated measures (MMRM). The model will include randomised arm (IPT-Work and SP), time point (post-treatment and follow-up), their interaction, continuous HRSD-24 baseline scores and their interaction with time point as fixed independent variables and the therapy group (cluster) as random effect. The model will allow for intra-subject correlation using a compound symmetry correlation structure. The two-sided test at significance level 5% for a difference between IPT-Work and SP at follow-up will be based on the two-sided 95% confidence interval derived for the corresponding difference in least-squares means estimated in the linear MMRM. The analysis assumes that missing values are missing at random (MAR), indicating that they are related to the independent variables, but not to the unmeasured outcomes themselves. No efficacy interim analysis will be performed. Except for the analysis concerning the primary estimand, all other statistical analyses will be considered as descriptive.

The difference of means between IPT-Work and SP with respect to the relative change in HRSD-24 score from baseline to post-treatment will be derived from the same linear MMRM as the primary estimand. For the binary outcomes based on the HRSD-24 score (response and remission), the analysis will be performed in a generalised linear mixed model with a logistic link function, under the assumption that missing values are MAR. For the further secondary estimands, change from baseline (difference post–pre) will be analysed in a linear MMRM. Additional details of the statistical analysis are specified in the full study protocol (see supplement) or will be provided in the statistical analysis plan.

## Discussion

This study protocol describes a large, multi-site RCT comparing a new type of a specific therapy (an adapted form of IPT focusing on work-related issues) with a nonspecific supportive intervention with a focus on broader issues in 144 patients with depression associated with work stress. Depression is one of the major causes for sick leaves and early retirements in Germany and problems at work contribute to the development and maintenance of depression. Depressive disorders have a major impact on occupational functioning and thus contributing not only to significant personal burden, but also to productivity loss and economic burden to organisations. Sickness absence and long-term work incapacity due to depression reached an all-time high in Germany [6] and other countries [31]. Since work-related stress has been described as the most common cause of depression by affected patients [32], targeted and effective treatment strategies for workplace depression are urgently needed.

“Work stress” was conceptualised as a novel focus of IPT, an evidence based depression intervention [33], addressing common psychosocial stressors at work. IPT-Work focuses on role stress and conflicts at work and the reduction of stressful working conditions. Findings of a preceding pilot study [11] comparing IPT-Work with standard treatment revealed IPT-Work to be more effective than TAU in reducing depression and work-related problems. Furthermore, the work-focused IPT programme turned out to be feasible and highly acceptable. It is hypothesised that IPT-Work is more effective not only in reducing depressive symptoms, but in increasing work ability and functioning compared to a supportive group approach 3 months after end of treatment.

Strengths of this study include meeting the requirements for a low risk of bias trial. Randomisation, blinding of raters, control of therapy allegiance and of overlapping treatments as well as for confounding factors are described and warranted by corresponding measures. The large sample size in combination with a multi-centre trial design allow the generalisation of the results. Using SP as a control group, applied in the same format, frequency, intensity, and duration as the IPT-work condition, enables an estimate of the specific effects of IPT-Work beyond the common therapy effects.

### Limitations

According to the German National Disease Management Guideline on Unipolar Depression [34] patients with moderate depressive episodes should be offered psychotherapy or drug treatment as equally effective alternatives, for patients with severe depressive episodes combined drug treatment and psychotherapy is recommended. However, to compare both forms of psychotherapy in our study with a medication or combination condition would require exorbitant sample sizes, but could be done in a next step. Another limitation of this study is the short follow-up period of only 3 months due to funding restrictions. Yet, a 12-months naturalistic follow-up is planned for a second study phase. Another possible critical point of the study design is that the same therapists deliver both forms of psychotherapy which aims at controlling for therapists effects on treatment outcome. However, the crossed therapist design could also be subject to bias due to differential psychotherapist allegiance. To evaluate this possible bias psychotherapist allegiance will be measured. A further limitation can be seen in the cluster-randomisation: Since both groups will not be randomized at the same point in time, seasonal effects cannot be excluded.

The goal of this study is to find ways to reduce psychosocial stress at work and thereby promote mental health and reduce the incidence of occupational depression. The outcomes of this trial could have meaningful societal implications for a large high risk population of work-stressed employees.

## Supplementary Information

Supplementary Material 1.

## Data Availability

No datasets were generated or analysed during the current study.

## Abbreviations

AE: Adverse Event
BDI: Beck Depression Inventory
B.Sc.: Bachelor of Science
CD-RISC: Connor-Davidson Resilience Scale
CTU: Clinical Trials Unit
BDI-II: Beck Depression Inventory-II
DFG: Deutsche Forschungsgemeinschaft (German Research Foundation)
DRKS: Deutsches Register Klinische Studien (German Clinical Trials Register)
DSM: Diagnostic and Statistical Manual of Mental Disorders
DSMB: Data Safety Monitoring Board
e.g.: For example
eCRF: Electronic Case Report Form
ERI: Effort-Reward-Imbalance
GCP: Good Clinical Practice
HRSD: Hamilton Rating Scale for Depression
ICH: International Council for Harmonisation of Technical Requirements for Pharmaceuticals for Human Use
IPT: Interpersonal psychotherapy
JCQ2: Job Content Questionnaire 2
M: Mean
MAR: Missing at random
MDD: Major Depressive Disorder
MMRM: Mixed model for repeated measures
No.: Number
ODI: Occupational Depression Inventory
QOL: Quality of Life
R: Revision
RCT: Randomised Controlled Trial
RTW-SE: Return-to-Work Self-efficacy (Questionnaire)
SAE: Serious Adverse Event
SCID: Structured Clinical Interview for DSM Disorders
SD: Standard deviation
SP: Supportive Psychotherapy
TAU: Treatment As Usual
V: Visit
Vs: Versus
W: Week(s)
WAI: Work Ability Index
WHO: World Health Organisation

## References

[1] World Employment and Social Outlook. Trends 2022. Geneva: International Labour Office; 2022.

[2] Roesler U, Jacobi F, Rau R. Work and mental disorders in a German national representative sample. Work Stress. 2006;20(3):234–44.

[3] Kessler RC, Merikangas KR, Wang PS. The prevalence and correlates of workplace depression in the national comorbidity survey replication. J Occup Environ Med. 2008;50(4):381. 18404010 10.1097/JOM.0b013e31816ba9b8 PMC2742688

[4] Mack S, Jacobi F, Beesdo-Baum K, Gerschler A, Strehle J, Höfler M, et al. Functional disability and quality of life decrements in mental disorders: results from the mental health module of the german health interview and examination survey for adults (Degs1-Mh). Eur Psychiatry. 2015;30(6):793–800. 26169476 10.1016/j.eurpsy.2015.06.003

[5] Angerer P, Gündel H, Kröger C, Rothermund E. Rationale, models, and impact of workplace-based psychotherapeutic services. Bundesgesundheitsblatt Gesundheitsforschung Gesundheitsschutz. 2024;67(7):743–50. 38806746 10.1007/s00103-024-03892-8 PMC11231002

[6] Dehl T, Hildebrandt S, Zich K, Nolting HD. Gesundheitsreport 2024. In: Storm A, Nürnberg V, editors. Beiträge zur Gesundheitsökonomie und Versorgungsforschung. Hamburg: medhochzwei Verlag GmbH; 2024.

[7] du Prel JB, Koscec Bjelajac A, Franić Z, Henftling L, Brborović H, Schernhammer E, et al. The relationship between work-related stress and depression: a scoping review. Public Health Rev. 2024;45:1606968. 38751606 10.3389/phrs.2024.1606968 PMC11094281

[8] Modini M, Joyce S, Mykletun A, Christensen H, Bryant RA, Mitchell PB, et al. The mental health benefits of employment: results of a systematic meta-review. Australas Psychiatry. 2016;24(4):331–6. 26773063 10.1177/1039856215618523

[9] Nieuwenhuijsen K, Verbeek JH, Neumeyer-Gromen A, Verhoeven AC, Bültmann U, Faber B. Interventions to improve return to work in depressed people. Cochrane Database Syst Rev. 2020;10:CD006237. 10.1002/14651858.CD006237.pub4 PMC8094165 33052607

[10] Cuijpers P, Andersson G, Donker T, van Straten A. Psychological treatment of depression: results of a series of meta-analyses. Nord J Psychiatry. 2011;65(6):354–64. 21770842 10.3109/08039488.2011.596570

[11] Schramm E, Mack S, Thiel N, Jenkner C, Elsaesser M, Fangmeier T. Interpersonal psychotherapy vs. treatment as usual for major depression related to work stress: a pilot randomized controlled study. Front Psychiatry. 2020;11:193. 32256410 10.3389/fpsyt.2020.00193 PMC7093578

[12] Niedermoser DW, Kalak N, Kiyhankhadiv A, Brand S, Walter C, Schweinfurth N, et al. Workplace-related interpersonal group psychotherapy to improve life at work in individuals with major depressive disorders: a randomized interventional pilot study. Front Psychiatry. 2020;11:168. 32256402 10.3389/fpsyt.2020.00168 PMC7090238

[13] Yunus WMAWM, Musiat P, Brown JSL. Systematic review of universal and targeted workplace interventions for depression. Occup Environ Med. 2018;75(1):66–75. 29074553 10.1136/oemed-2017-104532

[14] Hamilton M. A rating scale for depression. J Neurol Neurosurg Psychiatry. 1960;23(1):56–62. 14399272 10.1136/jnnp.23.1.56 PMC495331

[15] Beck AT, Steer RA, Brown GK. Manual for the beck depression inventory-II. San Antonio: Psychological Corperation; 1996.

[16] Tuomi K, Ilmarinen J, Jahkola A. Work ability index. Helsinki: Finnish Institute of Occupational Health; 1998.

[17] Lagerveld SE, Blonk RWB, Brenninkmeijer V, Schaufeli WB. Return to work among employees with mental health problems: development and validation of a self-efficacy questionnaire. Work Stress. 2010;24(4):359–75.

[18] Siegrist J, Starke D, Chandola T, Godin I, Marmot M, Niedhammer I, et al. The measurement of effort–reward imbalance at work: European comparisons. Soc Sci Med. 2004;58(8):1483–99. 14759692 10.1016/S0277-9536(03)00351-4

[19] Markowitz JC. What is supportive psychotherapy? Focus. 2014;12(3):285–9.

[20] Cuijpers P, Miguel C, Ciharova M, Harrer M, Karyotaki E. Non-directive supportive therapy for depression: a meta-analytic review. J Affect Disord. 2024;349:452–61. 38211757 10.1016/j.jad.2024.01.073

[21] Beesdo-Baum K, Zaudig M, Wittchen HU. Strukturiertes Klinisches Interview für DSM-5 [The Structured Clinical Interview for DSM-5-Clinician Version] (SCID-5-CV). Göttingen: Hogrefe; 2019.

[22] Bianchi R, Schonfeld IS. The occupational depression inventory: a new tool for clinicians and epidemiologists. J Psychosom Res. 2020;138:110249. 32977198 10.1016/j.jpsychores.2020.110249

[23] Rush AJ, Trivedi MH, Carmody TJ, Ibrahim HM, Markowitz JC, Keitner GI, et al. Self-reported depressive symptom measures: sensitivity to detecting change in a randomized, controlled trial of chronically depressed, nonpsychotic outpatients. Neuropsychopharmacol. 2005;30(2):405–16. 10.1038/sj.npp.1300614 15578008

[24] Cuijpers P, Driessen E, Hollon SD, van Oppen P, Barth J, Andersson G. The efficacy of non-directive supportive therapy for adult depression: a meta-analysis. Clin Psychol Rev. 2012;32(4):280–91. 22466509 10.1016/j.cpr.2012.01.003

[25] Veal C, Tomlinson A, Cipriani A, Bulteau S, Henry C, Müh C, et al. Heterogeneity of outcome measures in depression trials and the relevance of the content of outcome measures to patients: a systematic review. Lancet Psychiatry. 2024;11(4):285–94. 38490761 10.1016/S2215-0366(23)00438-8

[26] Carrozzino D, Patierno C, Fava GA, Guidi J. The Hamilton rating scales for depression: a critical review of clinimetric properties of different versions. Psychother Psychosom. 2020;89(3):133–50. 32289809 10.1159/000506879

[27] Karasek R, Brisson C, Kawakami N, Houtman I, Bongers P, Amick B. The Job Content Questionnaire (JCQ): An instrument for internationally comparative assessments of psychosocial job characteristics. J Occup Health Psychol. 1998;3(4):322–55. 10.1037//1076-8998.3.4.322 9805280

[28] The WHOQOL Group. Development of the World Health Organization WHOQOL-BREF quality of life assessment. Psychol Med. 1998;28(3):551–8. 9626712 10.1017/s0033291798006667

[29] Connor KM, Davidson JR. T. Development of a new resilience scale: The Connor-Davidson resilience scale (CD-RISC). Depress Anxiety. 2003;18:76–82. 10.1002/da.10113 12964174

[30] International Council for Harmonisation of Technical Requirements for Pharmaceuticals for Human Use (ICH). ICH E9 (R1) Addendum on Estimands and Sensitivity Analysis in Clinical Trials to the Guideline on Statistical Principles for Clinical Trials. https://www.ema.europa.eu/en/ich-e9-statistical-principles-clinical-trials-scientific-guideline. [cited: 2025-01-30].

[31] Plana-Ripoll O, Weye N, Knudsen AK, Hakulinen C, Madsen KB, Christensen MK, et al. The association between mental disorders and subsequent years of working life: a Danish population-based cohort study. Lancet Psychiatry. 2023;10(1):30–9. 36480953 10.1016/S2215-0366(22)00376-5

[32] Hansson M, Chotai J, Bodlund O. Patients’ beliefs about the cause of their depression. J Affect Disord. 2010;124(1):54–9. 19923007 10.1016/j.jad.2009.10.032

[33] Cuijpers P, Donker T, Weissman MM, Ravitz P, Cristea IA. Interpersonal psychotherapy for mental health problems: a comprehensive meta-analysis. Am J Psychiatry. 2016;173(7):680–7. 27032627 10.1176/appi.ajp.2015.15091141

[34] Bundesärztekammer (BÄK), Kassenärztliche Bundesvereinigung (KBV), Arbeitsgemeinschaft der Wissenschaftlichen Medizinischen Fachgesellschaften (AWMF). Nationale VersorgungsLeitlinie Unipolare Depression – Langfassung, Version 3.2. 2022. 10.6101/AZQ/000505. www.leitlinien.de/depression. [cited: 2025–01–30].

