# Supplementary material for: Interpersonal vs. supportive group psychotherapy for depression attributed to work stress: study protocol of the multicentre, cluster-randomised, controlled IPT-Work trial
Source: BMC Psychiatry. 2025 Feb 19;25:149. doi: 10.1186/s12888-025-06594-w (PMC11841184; doi:10.1186/s12888-025-06594-w)
Supplement: Supplementary file 1 — Supplementary Material 1. [file 12888_2025_6594_MOESM1_ESM.pdf]

PROTOCOL

An interpersonal group psychotherapy for depression and work stress versus supportive psychotherapy: a multicentre, randomised, controlled trial

IPT-Work

Version 1.1

27th January 2025

Protocol History: V 1.0 / 26.11.2024

Coordinating Investigator
Prof. Dr. Elisabeth Schramm
Department of Psychiatry and Psychotherapy
Medical Center - University of Freiburg
Hauptstr. 5
79104 Freiburg

Tel. +49 (0) 761 270 69670
elisabeth.schramm@uniklinik-freiburg.de

This Protocol contains confidential information. Circulation of this material to individuals who are not involved in the carrying out of the register or any kind of publication requires the approval of the Coordinator. These limitations similarly relate to all confidential information and data which will be obtained in the future.

Approval of the Protocol:

Coordinating Investigator		
Prof. Dr. Elisabeth Schramm	Signature	Date
Biometrics/Statistics		
Dr. Erika Graf	Signature	Date

Amendment

Summary of changes to the protocol:

Chapter	Topic and reason for changes	Wording V1.0	Wording V1.1
List of Abbreviations	Additions in the list of Abbreviations		Abbreviations added:<br>ATC, B.Sc., CBT, M, RCT, TAU
3.2 Participating Centres	Adjustments in response to comments from the Ethics Committee	6 centres are planned in Germany, which must meet the structural and personnel requirements for performing the planned regular study-related investigations.	6 centres in Germany (2x Freiburg, Mannheim, Frankfurt am Main, Rosenheim/Prien and Hannover), which must meet the structural and personnel requirements for performing the planned regular study-related investigations, have been selected and initiated to carry out the trial:.<br>• Department of Psychiatry and Psychotherapy, Medical Center - University of Freiburg (PI: Prof Dr Elisabeth Schramm)<br>• Department of Psychosomatics, Medical Center - University of Freiburg (PI: Prof Dr Claas Lahmann)<br>• Department of Psychiatry and Psychotherapy, Central Institute of Mental Health, J5, Medical Faculty Mannheim, University of Heidelberg (PI: Prof Dr Michael Deuschle)<br>• Department of Psychiatry, Social Psychiatry and Psychotherapy, Hannover Medical School (PI: Prof Dr Kai Kahl)<br>• Department of Psychiatry, Psychosomatic Medicine and Psychotherapy, University Hospital Frankfurt – Goethe University Frankfurt (PI: Prof Dr Andreas Reif)<br>• Schoen Clinic Roseneck, Prien am Chiemsee (PI: Prof Dr Dr Andreas Hillert)
4 Subject Population and Selection Criteria	Adjustments in response to comments from the Ethics Committee	… into the trial.	… into the trial.<br>The recruitment procedure for this randomized controlled trial (RCT) is based on the successful procedure of our pilot study14. There, the recruitment process proved to be effective, even under more demanding conditions. Specifically, the pilot study required a larger sample size per centre (n=28), and participants in both groups had to be randomized simultaneously. Despite these challenges, we were able to recruit participants within the planned timeframe. This experience underscores the feasibility and robustness of our recruitment strategy, which has been slightly refined for the current RCT to further enhance its efficiency.
Table 5 Visit Schedule and chapter 5.1 Patient screening for eligibility	Addition		Work Context<br>Patient treatment preference

Chapter	Topic and reason for changes	Wording V1.0	Wording V1.1
Synopsis, 4.1 Inclusion Criteria, 5.2 Patient randomisation	precision regarding the reference date for the age of the examination results	day of randomisation	day of randomisation request
10.5.4 Trial interventions			…<br>Patients’ treatment preferences at baseline and post-treatment will be evaluated descriptively.
Complete document	refinement of the content		
Complete document	minor general revisions		

Investigator Statement

**Title:**	An interpersonal group psychotherapy for depression and work stress versus supportive psychotherapy. A multicentre, randomised, controlled trial (IPT-Work)
**Coordinating Investigator**	Prof. Dr. phil. Elisabeth Schramm
**Study Centre:**	
**Principal Investigator in the Centre:**	

I confirm that I have read the Study Protocol and hereby commit myself to adhere to all actions and terms as specified in the relevant sections of the clinical, ethical and general paragraphs.

I confirm that I and my colleagues will abide by the local legislation (in Germany). I further confirm that the Study will be carried out in compliance with the Declaration of Helsinki and ICH-GCP guidelines.

I acknowledge that all confidential information in this document will not be used or circulated without the prior written consent of the Coordinating Investigator.

I will put copies of this Study Protocol and possible updates at the disposal of my colleagues as well as ensure access to all information regarding the carrying out of this Study Protocol I will discuss this Study Protocol in detail with my colleagues and ensure that they are comprehensively informed about the trial compound/preparation and the execution of the study.

Furthermore I commit myself not to commence patient enrolment before the approval of the authorities and acceptance by the relevant/responsible Ethics Committee.

Date (DD/MM/YY) Signature of the Principal Investigator

List of Abbreviations

AE	Adverse Event
ATC	Anatomical Therapeutic Chemical Classification System
BDI	Beck Depression Inventory
B.Sc.	Bachelor Of Science
CBT	Cognitive Behavioral Therapy
CD-RISC	Connor-Davidson Resilience Scale
CI	Coordinating Investigator
CRA	Clinical Research Associate
CTU	Clinical Trials Unit
BDI-II	Beck Depression Inventory-II
DFG	Deutsche Forschungsgemeinschaft (German Research Foundation)
DRKS	Deutsches Register Klinische Studien (German Clinical Trials Register)
DSM	Diagnostic and Statistical Manual of Mental Disorders
DSMB	Data Safety Monitoring Board
e.g.	for example
eCRF	electronic Case Report Form
EDC	Electronic Data Capture
ERI	Effort-Reward-Imbalance
FAS	Full Analysis Set
FPFV	First Patient first Visit
FU	Follow Up
GCP	Good Clinical Practice
HRSD	Hamilton Rating Scale for Depression
ICC	Intra-cluster correlation
ICD	International Statistical Classification of Diseases and Related Health Problems
ICH	International Council for Harmonisation of Technical Requirements for Pharmaceuticals for Human Use
ID	identity
IPT	Interpersonal psychotherapy
JCQ2	Job Content Questionnaire 2
LPLV	Last Patient, last Visit
M	Mean
MAR	Missing at random
MD	Major Depression
MDD	Major Depressive Disorder
MedDRA	Medical Dictionary for Regulatory Activities
MMRM	Mixed model for repeated measures
MNAR	Missing not at random
No.	number
Non-start	Randomised treatment not started
ODI	Occupational Depression Inventory
PHI	Protected Health Information
PI	Pricipal Investigator
PT	MedDRA preferred term
Q	quarter
QOL	Quality of Life
R	revision
RCT	Randomised Controlled Trial
RTW-SE	Return-to-Work Self-efficacy (Questionnaire)
SAE	Serious Adverse Event
SAF	Safety set

SAP	Statistical Analysis Plan
SAS	Statistical Analysis System
SCI	Structured Clinical Interview
SCID	Structured Clinical Interview for DSM Disorders
SD	Standard deviation
SDV	Source Data Verification
SOC	MedDRA System Organ Class
SOP	Standard Operating Procedure
SP	Supportive Psychotherapy
TAU	Treatment As Usual
TMF	Trial Master File
V	Visit
Vs	versus
W	Week(s)
WAI	Work Ability Index
WHO	World Health Organization

Table of Contents

Approval of the Protocol: **2**
Amendment **3**
Investigator Statement **5**
List of Abbreviations **6**
Table of Contents **8**
List of Tables **10**
List of Figures **10**
Synopsis **11**
Synopse **13**
Responsibilities **15**
1 Introduction **17**
1.1 Background and Scientific Rationale 17
1.2 Own previous work 17
2 Objectives and Endpoints **18**
2.1 Definition of the primary estimand and full analysis set 21
2.2 Definition of secondary estimands 21
2.2.1 Secondary estimands based on the Hamilton Rating Scale for Depression 21
2.2.2 Further secondary estimands 21
2.3 Definition of the safety set 22
2.4 Estimands: Overview 22
3 Study Plan **23**
3.1 Study Design 23
3.2 Participating Centres 23
3.3 Number of Study Participants 24
3.4 Methods against bias 24
3.5 Roles and Responsibilities 24
3.5.1 Coordinating investigator 24
3.5.2 Principal investigators at the sites 25
3.5.3 Rater 25
3.5.4 Psychotherapists 25
4 Subject Population and Selection Criteria **25**
4.1 Inclusion Criteria 26
4.2 Exclusion Criteria 26
5 Procedures **27**
5.1 Patient screening for eligibility 29
5.2 Patient randomisation 30
5.3 Treatment 30
5.3.1 General procedures in both study arms 30
5.3.2 IPT-Work (intervention arm) 31

5.3.3 Supportive Psychotherapy (SP, control arm) 31
5.4 Follow-up evaluations 32
5.4.1 Blinding of raters 32
5.4.2 Post-treatment, Follow-up / End of Trial 32
6 Discontinuation criteria 32
6.1 Premature termination of one of the treatment arms or the entire study....................32
6.2 Premature termination of the study at one of the sites....................32
6.3 Discontinuation of study treatment or study participation for individual patients....................33
7 Safety monitoring and documentation 33
7.1 Definition of adverse events (AEs) and serious adverse events (SAEs) 33
7.2 Documentation of AEs and serious AEs (SAEs) 34
7.3 SAE reporting 34
7.4 Suicidality....................34
8 Data Collection and Management 35
8.1 Data confidentiality 35
8.2 Documentation of trial data....................35
8.2.1 Documentation in medical records 35
8.2.2 Documentation in (e)CRF 35
8.3 Data management 36
8.4 Data coding....................36
9 Quality Assurance System....................36
9.1 Quality Control (Monitoring)....................36
9.2 Source Data Verification (SDV) 37
9.3 Quality Assurance (Auditing) 37
10 Biostatistical Planning and Analysis 37
10.1 Trial Design....................37
10.2 Blinding of study statistician 38
10.3 Definition of estimands and analysis sets....................38
10.4 Sample size calculation 38
10.5 Methods of analysis 38
10.5.1 Descriptive statistics 38
10.5.2 Patient recruitment, disposition of patients 39
10.5.3 Patients‘ baseline characteristics 39
10.5.4 Trial interventions 39
10.5.5 Concomitant treatment and medication 39
10.5.6 Adherence of study therapists and interrater reliability 39
10.5.7 Analysis of primary estimand 40
10.5.8 Analysis of secondary estimands 40
10.5.9 Analysis of safety 41
11 Data Safety Monitoring Board (DSMB) 41
12 Ethical and Legal Principles 41
12.1 Subject Informed Consent 41

12.2 Ethical and Regulatory Requirements 42
12.3 Data Protection and Confidentiality 42
12.4 Archiving 42
13 Registry and Publications 42
14 Administrative agreements 43
14.1 Financing of the project 43
14.2 Study reports 43
15 References 43

List of Tables

Table 1: Objectives and endpoints 18
Table 2: Variables 21
Table 3: Overview of Estimands 22
Table 4: Timetable for the Study 23
Table 5: Visit Schedule 28

List of Figures

Figure 1: Study Design 23

Synopsis

STUDY TITLE	An interpersonal group psychotherapy for depression and work stress versus supportive psychotherapy: a multicentre, randomised, controlled trial
**ABBREVIATED TITLE**	IPT-Work
**TRIAL REGISTER(S)**	DRKS00035259<br>FRKS005515
**PROTOCOL NUMBER**	P003344
**INDICATION/ MAIN DIAGNOSIS**	Major Depression
**STUDY OBJECTIVES**	To investigate the efficacy of a specific interpersonal group psychotherapy for depression and work stress (IPT-Work) to reduce depressive symptoms and increase work ability compared to a non-specific supportive group psychotherapy (SP).
**STUDY DESIGN**	Interventional, multicentre, cluster-randomised, active-controlled, observer-blinded clinical trial with two parallel groups (therapeutic confirmatory)
**TREATMENTS / PROCEDURES**	**Experimental intervention**: A manual-based interpersonal group intervention with a specific focus on the work context of 10 sessions over 8 weeks (twice weekly in the first 2 weeks and weekly thereafter) of 90 minutes duration for 4 to 6 outpatients<br>**Control intervention:** A manual-based non-specific supportive group intervention (SP) of 10 sessions over 8 weeks (twice weekly in the first 2 weeks and weekly thereafter) of 90 minutes duration for 4 to 6 outpatients<br>**Follow-up per patient:** 5 months after randomisation<br>**Duration of intervention per patient:** 8 weeks
**ENDPOINTS**	**Primary endpoint:** Relative change in HRSD-24 score from baseline to follow-up 3 months after end of treatment (100 – (100 x post/pre) %); IPT-Work vs. SP<br>**Secondary endpoint(s):** Relative change in HRSD-24 score from baseline to post-treatment 9 weeks after randomisation; Occupational Depression Inventory (ODI), Beck Depression Inventory-II (BDI-II), Work Ability Index (WAI), Return to Work Attitude (RTW-SE), Effort-Reward-Imbalance (ERI), Job Content Questionnaire 2 (JCQ2), Quality of Life (WHOQOL-BREF), and Connor-Davidson Resilience Scale (CD-RISC) at post-treatment and follow-up. In addition, remission (HRSD-24 <9) and response (reduction in the HRSD-24 score by at least 50% from baseline) rates at post-treatment and follow-up. Days of sick leave throughout the study period.<br>**Assessment of safety:** Frequency of (serious) adverse events (AEs and SAEs) reported by treatment arm.
**TIMETABLE**	Start of Study: Q4/2024<br>Funding period: 01.05.2024 – 30.04.2027
**SAMPLE SIZE**	144
**STATISTICAL ANALYSIS**	**Efficacy:** With a two-sided 5% level two-group t-test, a sample size of 124 observations yields 80% power to detect a difference if HRSD-24 mean relative changes from baseline differ by 28.0% points, assuming a common SD of 55.0%. Due to intra-cluster correlation and missing follow-up data, 144 patients should be randomised.<br>**Description of the primary efficacy analysis and population**: Effects of treatment will be analysed with a linear mixed model for repeated measures including randomised arm, time point and their interaction as well as HRSD-24 baseline scores and their interaction with time point as fixed effects. The model will allow for intracluster and intrasubject correlation. The treatment effect will be tested based on the two-sided 95% confidence interval derived from the difference in least-squares means estimated in the mixed model.<br>**Safety:** The incidences of AEs and SAEs will be summarised by treatment arm with corresponding two-sided 95% confidence intervals.<br>**Secondary endpoints:** will be analysed descriptively in a similar fashion as the primary outcome, using regression models as appropriate for the respective type of data. Treatment effects: estimated with two-sided 95% confidence intervals.
**INCLUSION CRITERIA**	1. Patient's written informed consent has been obtained<br>2. Primary diagnosis of Major Depression/MD (single-episode or recurrent) according to the Structured Clinical Interview for DSM-5 (SCID-5-CV; also ICD-10 coded; assessment not older than 14 days on the day of randomisation request)<br>3. A score of ≥ 17 on the 24-item version of the Hamilton Rating Scale for Depression (HRSD-24; assessment not older than 14 days on the day of randomisation request)<br>4. A total score of at least 15 on the Occupational Depression Inventory (ODI; assessment not older than 14 days on the day of randomisation request)<br>5. Sick leave related to depressive or burn-out complaints for at least 7 days during the last 12 months

	6. Sufficient German language skills<br>7. Outpatient status<br>8. 18-years or older<br>9. Patients must have a regular work activity that is expected to continue for at least 6 months at the time of randomisation.
**EXCLUSION CRITERIA**	1. Acute risk of suicide<br>2. History of psychotic symptoms, bipolar disorder, or organic brain disorders<br>3. A primary diagnosis of another SCID-5-CV disorder<br>4. Concurrent diagnosis of substance dependency<br>5. Antisocial, schizotypal, or borderline personality disorder (SCID-5-PD)<br>6. Other ongoing psychotherapy<br>7. Antidepressive pharmacotherapy (if not stable for at least the last 4 weeks before randomisation)<br>8. Serious medical condition or time restrictions interfering with participation in regular sessions<br>9. Current sick leave > 4 weeks<br>10. Applying for rehabilitation or early retirement<br>11. Patient without legal capacity who is unable to understand the nature, significance and consequences of the study<br>12. Simultaneous participation in other studies which could interfere with this study and/or participation before the end of a required restriction period. No prior randomisation in this IPT-Work trial.<br>13. Persons who are in a disciplinary employment relationship with a member of the study team

Synopse

STUDIENTITLE	Ein interpersonelles Gruppenprogramm für Depression und Arbeitsstress (IPT-Work) versus supportive Psychotherapie: Eine multizentrische, randomisiert-kontrollierte Studie
**KURZTITEL**	IPT-Work
**STUDIENREGISTER**	DRKS00035259<br>FRKS005515
**INDIKATION/ HAUPTDIAGNOSE**	Depression
**STUDIENZIELE**	Untersuchung der Wirksamkeit einer spezifischen interpersonellen Gruppenpsychotherapie für Depression und Arbeitsstress (IPT-Work) zur Verringerung depressiver Symptome und zur Verbesserung der Arbeitsfähigkeit im Vergleich zu einer unspezifischen unterstützenden Gruppenpsychotherapie (SP).
**STUDIENDESIGN**	Interventionelle, multizentrische, cluster-randomisierte, aktiv-kontrollierte, beobachterverblindete klinische Studie mit zwei parallelen Gruppen (konfirmatorisch zur Therapiebestätigung)
**BEHANDLUNG**	**Experimentelle Intervention:** Eine manualbasierte interpersonelle Gruppenintervention mit besonderem Fokus auf den Arbeitskontext mit 10 Sitzungen über 8 Wochen (zweimal wöchentlich in den ersten 2 Wochen und danach wöchentlich) von 90 Minuten Dauer für 4 bis 6 ambulante Patienten<br>**Kontrollintervention:** Eine manualbasierte unspezifische unterstützende Gruppenintervention (SP) von 10 Sitzungen über 8 Wochen (zweimal wöchentlich in den ersten 2 Wochen und danach wöchentlich) von 90 Minuten Dauer für 4 bis 6 ambulante Patienten<br>**Follow-up pro Patient:** 5 Monate nach Randomisierung<br>**Dauer der Intervention pro Patient:** 8 Wochen
**ENDPUNKTE**	**Primärer Endpunkt:** Relative Veränderung des HRSD-24-Wertes vom Ausgangswert bis zur Nachuntersuchung 3 Monate nach Behandlungsende (100 - (100 x post/pre) %); IPT-Work vs. SP<br>**Sekundäre(r) Endpunkt(e):** Relative Veränderung des HRSD-24-Wertes vom Ausgangswert bis zur Nachbeobachtung 9 Wochen nach der Randomisierung; Occupational Depression Inventory (ODI), Beck Depression Inventory-II (BDI-II), Work Ability Index (WAI), Return to Work Attitude (RTW-SE), Effort-Reward-Imbalance (ERI), Job Content Questionnaire 2 (JCQ2), Quality of Life (WHOQOL-BREF) und Connor-Davidson Resilience Scale (CD-RISC) nach der Behandlung und bei der Nachuntersuchung. Außerdem: Remissions- (HRSD-24 <9) und Ansprechraten (Verringerung des HRSD-24-Scores um mindestens 50 % gegenüber dem Ausgangswert) nach der Behandlung und bei der Nachuntersuchung. Krankenstandstage während des gesamten Studienzeitraums.<br>**Bewertung der Sicherheit:** Häufigkeit von (schwerwiegenden) unerwünschten Ereignissen (AEs und SAEs), die je Behandlungsarm gemeldet wurden.
**ZEITPLAN**	Start der Studie: Q4/2024<br>Förderperiode: 01.05.2024 – 30.04.2027
**FALLZAHL**	144
**STATISTISCHE ANALYSE**	**Wirksamkeit:** Mit einem zweiseitigen unabhängigen t-Test auf dem 5%-Niveau ergibt sich bei einer Stichprobengröße von 124 Beobachtungen eine statistische Power von 80% zum Nachweis eines Unterschiedes, wenn sich die mittleren relativen Veränderungen des HRSD-24 gegenüber dem Ausgangswert um 28,0%-Punkte unterscheiden und eine gemeinsame Standardabweichung von 55,0% angenommen wird. Aufgrund der Intra-Cluster-Korrelation und fehlender Follow-up-Daten sollen 144 Patienten randomisiert werden.<br>**Beschreibung der primären Wirksamkeitsanalyse und der Population:** Die Behandlungseffekte werden mit einem linearen gemischten Modell für wiederholte Messungen analysiert, das den randomisierten Arm, den Zeitpunkt und deren Wechselwirkung sowie die HRSD-24-Basisscores und deren Wechselwirkung mit dem Zeitpunkt als feste Effekte berücksichtigt. Dabei werden Intra-Cluster und Intra-Subjekt-Korrelation berücksichtigt. Der Behandlungseffekt wird auf der Grundlage des zweiseitigen 95%-Konfidenzintervalls für den Kleinste-Quadrate-Schätzers der Mittelwertdifferenz aus dem gemischten Modells getestet.<br>**Sicherheit:** Die Inzidenzen von UE und SUE werden nach Behandlungsarm mit den entsprechenden zweiseitigen 95%-Konfidenzintervallen zusammengefasst.<br>**Sekundäre Endpunkte:** werden in ähnlicher Weise deskriptiv analysiert wie der primäre Endpunkt, wobei Regressionsmodelle verwendet werden, die für die jeweilige Art von

	Daten geeignet sind. Behandlungseffekte: geschätzt mit zweiseitigen 95%-Konfidenzintervallen.
**EINSCHLUSSKIRITERIEN**	1. Die schriftliche Einwilligung des Patienten nach Aufklärung liegt vor<br>2. Hauptdiagnose Depression (einmalige oder rezidivierende Depression) gemäß dem Structured Clinical Interview for DSM-5 (SCID-5-CV; auch ICD-10 kodiert; Bewertung nicht älter als 14 Tage am Tag der Randomisierungsanforderung)<br>3. Ein Ergebnis von ≥ 17 auf der 24-teiligen Version der Hamilton Rating Scale for Depression (HRSD-24; Bewertung nicht älter als 14 Tage am Tag der Randomisierungsanforderung)<br>4. Ein Gesamtwert von mindestens 15 auf dem Occupational Depression Inventory (ODI; Bewertung nicht älter als 14 Tage am Tag der Randomisierungsanforderung)<br>5. Mindestens 7 Krankenstandstage aufgrund von depressiven oder Burn-out-Beschwerden während der letzten 12 Monate<br>6. Ausreichende deutsche Sprachkenntnisse<br>7. Ambulanter Status<br>8. Mindestalter von 18 Jahre oder älter<br>9. Die Patienten müssen zum Zeitpunkt der Randomisierung einer regelmäßigen beruflichen Tätigkeit nachgehen, die voraussichtlich für mindestens 6 Monate andauern wird.
**AUSSCHLUSSKRITIERIEN**	1. Akute Selbstmordgefährdung<br>2. Psychotische Symptome, bipolare Störung oder organische Hirnstörungen in der Vorgeschichte<br>3. Eine Primärdiagnose einer anderen SCID-5-CV-Störung<br>4. Gleichzeitige Diagnose einer Substanzabhängigkeit<br>5. Antisoziale, schizotypische oder Borderline-Persönlichkeitsstörung (SCID-5-PD)<br>6. Andere laufende Psychotherapie<br>7. Antidepressive Pharmakotherapie (falls nicht mindestens in den letzten 4 Wochen vor der Randomisierung stabil)<br>8. Schwerwiegender medizinischer Zustand oder zeitliche Einschränkungen, die die Teilnahme an regelmäßigen Sitzungen behindern<br>9. Aktueller Krankenstand > 4 Wochen<br>10. Antrag auf Rehabilitation oder Vorruhestand<br>11. Nicht geschäftsfähiger Patient, der nicht in der Lage ist, die Art, Bedeutung und Folgen der Studie zu verstehen<br>12. Gleichzeitige Teilnahme an anderen Studien, die diese Studie beeinträchtigen könnten, und/oder Teilnahme vor Ablauf einer erforderlichen Sperrfrist. Keine vorherigen Randomiserung in dieser IPT-Work Studie.<br>13. Personen, die in einem abhängigen Arbeitsverhältnis mit einem Mitglied des Studienteams stehen

Responsibilities

**Coordinating Investigator**	Name:	Prof. Dr. Elisabeth Schramm
	Institution:	Department of Psychiatry and Psychotherapy<br>Medical Center – University of Freiburg
	Address:	Hauptstr. 5<br>79104 Freiburg, GERMANY
	Telephone:	+49 (0) 761 270-69670
	E-Mail:	elisabeth.schramm@uniklinik-freiburg.de
**Biostatistician**	Name:	Dr. Erika Graf
	Institution:	Medical Center – University of Freiburg,<br>Institute of Medical Biometry and Statistics
	Address:	Stefan-Meier-Str. 26, 79104 Freiburg, GERMANY
	Telephone:	+49 761 270-83743
	E-Mail:	erika.graf@uniklinik-freiburg.de
**Randomisation**	Institution:	Medical Center – University of Freiburg, Clinical Trials Unit
	Address:	Elsaesser Str. 2, 79110 Freiburg, GERMANY
	Fax:	+49 761 270-74390
	E-Mail:	zks.dm@list.uniklinik-freiburg.de
**Project Manager (PM)**	Name:	Johannes Bausch
	Institution:	Medical Center – University of Freiburg, Clinical Trials Unit
	Address:	Elsaesser Str. 2, 79110 Freiburg, GERMANY
	Telephone:	+49 761 270-27630
	Fax:	+49 761 270-74250
	E-Mail:	johannes.bausch@uniklinik-freiburg.de
**Monitoring (CRA(s))**	Institution:	Medical Center – University of Freiburg, Clinical Trials Unit
	Address:	Elsaesser Str. 2, 79110 Freiburg, GERMANY
	Telephone:	+49 761 270-74050
	Fax:	+49 761 270-73730
	E-Mail	zks.mo@list.uniklinik-freiburg.de
**Data management (DM)**	Institution:	Medical Center – University of Freiburg, Clinical Trials Unit
	Address:	Elsaesser Str. 2, 79110 Freiburg, GERMANY
	Telephone:	+49 761 270-77109
	Fax:	+49 761 270-74390
	E-Mail:	zks.dm@list.uniklinik-freiburg.de
**SAE reporting**	Name:	Prof. Dr. Elisabeth Schramm
	Institution:	Department of Psychiatry and Psychotherapy<br>Medical Center – University of Freiburg
	Address:	Hauptstr. 5, 79104 Freiburg, GERMANY
	Telephone:	+49 (0) 761 270-69670
	E-Mail:	elisabeth.schramm@uniklinik-freiburg.de

**Data Monitoring Committee**	Name:	Prof. Dr. Johannes Siegrist
	Institution:	Institute of Medical Sociology, Universität Düsseldorf
	Profession:	Professor emeritus of Medical Sociology
	Address:	Merowingerplatz 1a, 40225 Düsseldorf, GERMANY
	Telephone:	+49 211 81-06008
	E-mail:	siegrist@uni-duesseldorf.de
	Name:	Prof. Dr. Jan Philipp Klein
	Institution:	Department of Psychiatry and Psychotherapy, University of Lübeck
	Profession:	Senior Consultant in Psychosomatics and Psychotherapy
	Address:	Ratzeburger Allee 160; 23538 Lübeck, GERMANY
	Telephone:	+49 451 500-98840
	Fax:	+49 451 500-98844
	E-mail:	Philipp.Klein@uksh.de
	Name:	Dr. Marietta Kirchner
	Institution:	Institute of Medical Biometry and Statistics, University of Heidelberg
	Profession:	Statistician
	Address:	Im Neuenheimer Feld 130.3, 69120 Heidelberg, GERMANY
	Telephone:	+49 6221 56-7784
	Fax:	+49 6221 56-4195
	E-mail:	kirchner@imbi.uni-heidelberg.de

1 Introduction

1.1 Background and Scientific Rationale

Unipolar depression is highly prevalent at the workplace with every 10th female and every 20th male worker meeting criteria for major depression[1,2]. The prevalence of major depressive disorder (MDD) among employees has been estimated at 7.6%[3]. Depressive disorders have a major impact on social and occupational functioning[4] and are increasingly recognised as a significant mental health problem in the workplace contributing to productivity loss and economic burden to organisations[5]. MDD are among the leading causes of sick leave and long-term work incapacity in most modern countries. Administrative data from national health statistics document a fourfold increase in days of sick leave due to mental disorders, particularly depression, between 1997 and 2023[6]. Work-related stress has been described as the most common cause of depression by patients[7]. In a scoping review, the majority of the 125 included studies report significant associations between work-related stress and depression[8]. At the same time, the positive effects of good work and the role work can play in facilitating recovery from an illness and enhancing mental well-being [9], need to be highlighted and adressed in psychotherapeutic treatment.

Novel aspect of the proposed trial: There is an urgent need to evaluate specific innovative treatments for depression and work stress by adapting existing interventions to focus on the work context and to include work-related outcomes[10]. The best investigated predictors for depression in the context of work stress are psychosocial in nature and include high job demands in connection with low decision latitude (demand-control-imbalance), low social support, lack of gratification (effort-reward-imbalance), interpersonal conflicts, role stress, and organisational injustice[11]. For the specific therapy of workplace depression, a novel focus “work stress” of Interpersonal Psychotherapy (IPT-Work[12]) was conceptualised addressing those psychosocial stressors as work usually takes place in an interpersonal context. Interpersonal Psychotherapy (IPT) is a first line treatment for depression[13] whose effectiveness has been demonstrated for the four standard foci “interpersonal disputes”, “role transitions”, “social deficits”, and “grief”. There is preliminary evidence[14,15] that the additional focus of “work-stress” (IPT-Work) is an appropriate fit for the therapy of occupational problems associated with the depressive episode.

Clinical impact: New findings suggest that workplace directed interventions facilitate the recovery of employees diagnosed with MDD and produce beneficial effects on occupational outcomes. However, the small number of controlled studies on the effects of psychotherapy (with or without antidepressant medication) on work-related outcomes in depression makes it difficult to draw final conclusions. Our primary hypothesis is that IPT-Work is more effective in reducing depressive symptoms and in increasing work ability compared to SP 3 months after end of treatment.

1.2 Own previous work

In our pilot study, we evaluated the feasibility and generated first data on the effectiveness of Interpersonal Psychotherapy (IPT) adapted as a group psychotherapy to focus on the work context (W-IPT). In total, 28 outpatients (22 women; M = 49.8 years old) with Major Depressive Disorder related to work stress were randomised to 8 weekly group sessions of W-IPT or to treatment as usual (TAU; guideline oriented treatment). Primary endpoint was the Hamilton Rating Scale for Depression (HRSD-24) score. Key secondary endpoints were, among others, Beck Depression Inventory (BDI-II), Work Ability Index (WAI), Return to Work Attitude (RTW-SE), and the Effort-Reward-Imbalance (ERI). In addition, we evaluated the participants' overall satisfaction with the W-IPT programme by two items. A follow-up assessment was conducted 3 months after end of acute treatment. W-IPT was significantly more effective than TAU in reducing clinician-assessed depressive symptoms at follow-up (HRSD-24 W-IPT/TAU: M = 6.6/12.0, SE: 1.46/2.17,

t(df = 1) = −2.24, p = 0.035, d = 0.79) and self-assessed depression (BDI-II W-IPT/TAU post-treatment: M = 8.8/18.8, SE: 1.69/2.70, t(df = 1) = −3.82, p = 0.001, d = 1.28; follow-up: M = 8.8/16.1, SE: 1.62/2.26, t(df = 1) = −2.62, p = 0.015, d = 0.99). Furthermore, W-IPT was superior in improving work-ability (WAI), return-to-work attitude (RTW-SE), and the effort-reward-ratio (ERI). No dropouts were observed in both groups. The vast majority (89 percent) of participants in the W-IPT condition were "very satisfied" with the programme, although wishing for a greater number of sessions (75 percent). A work-focused IPT programme for the treatment of depression associated to work stress was feasible and highly acceptable. W-IPT turned out to be more effective than standard treatment in reducing depression and work-related problems. However, further evidence in a multicentre trial implementing a standardised control arm is necessary.

We discarded TAU (defined as usual treatment/routine care according to accepted standards; might include pharmacotherapy and/or psychotherapy) as a control condition for this study and use Supportive group Psychotherapy (SP)[16] instead since a TAU group might be very heterogeneous and is susceptible to bias. With TAU, the treatment in the experimental group would be much more intense than the control group. Therefore, the study might suffer from performance bias and it could not be distinguished if the observed effect is caused by the treatment itself or simply by the different treatment intensities. By using SP, treatment intensity is equal, and in both conditions no other concurrent psychotherapy outside the study participation is allowed.

SP is a manual-based[16], nonspecific, non-work-directed psychotherapeutic intervention found to be effective in the treatment of depression[17]. SP will be applied in the same format (group sessions), frequency, intensity, and duration as the IPT-work condition in order to implement a homogeneous and comparable control group. Since SP is based on exclusively common therapy effects (common factors approach) and IPT-Work elicits common as well as specific therapy effects, using SP as control group enables an estimate of the specific effects of IPT-Work beyond the common therapy effects. This is not possible in a comparison with another "treatment package" like Cognitive Behavioral Therapy (CBT) implementing both specific and common therapy effects as well. In line with this, SP has proven useful and feasible as control group in a variety of rigorous multicentre psychotherapy RCTs, e.g. Schramm et al.[18] or Dafsari et al.[19,20].

2 Objectives and Endpoints

The following table describes the objectives of the trial and the endpoints used for meeting the objectives. The corresponding estimands will be defined and described in sections 2.1, 2.2, and 2.3 and summarised in section 2.4.

Table 1: Objectives and endpoints

Estimand	No.	Objective	Endpoint (Variable in estimand)
**Primary Estimand** (see section 2.1)	1	To assess the efficacy of IPT-Work in comparison to SP on <u>depression severity at follow-up</u>	Relative change in HRSD-24 score from baseline to follow-up 3 months after end of treatment (100 – (100 x post/pre) %)
**Secondary Estimands** (see section 2.2)	2	To assess the efficacy of IPT-Work in comparison to SP on <u>depression severity at post-treatment</u>	Relative change in HRSD-24 score from baseline to post-treatment 9 weeks after randomisation (100 – (100 x post/pre) %)

Estimand	No.	Objective	Endpoint (Variable in estimand)
**Secondary Estimands** (see section 2.2)	3	To assess the efficacy of IPT-Work in comparison to SP on the frequency of remission at post-treatment	Remission, defined as HRSD-24 score of < 9 (yes/no) at end post-treatment 9 weeks after randomisation
**Secondary Estimands** (see section 2.2)	4	To assess the efficacy of IPT-Work in comparison to SP on the frequency of remission at follow-up	Remission, defined as HRSD-24 score of < 9 (yes/no) at follow-up 3 months after end of treatment
**Secondary Estimands** (see section 2.2)	5	To assess the efficacy of IPT-Work in comparison to SP on the frequency of response at post-treatment	Response, defined as the reduction in the HRSD-24 score by at least 50% from baseline (yes/no) at post-treatment 9 weeks after randomisation
**Secondary Estimands** (see section 2.2)	6	To assess the efficacy of IPT-Work in comparison to SP on the frequency of response at follow-up	Response, defined as the reduction in the HRSD-24 score by at least 50% from baseline (yes/no) at follow-up 3 months after end of treatment
**Secondary Estimands** (see section 2.2)		To assess the efficacy of IPT-Work in comparison to SP on<br>• Self-rated work-attributed depressive symptoms<br>• Self-rated depressive symptoms<br>• Self-rated ability to work<br>• Self-rated return-to-work self-efficacy<br>• Self-rated effort-reward imbalance<br>• Self-rated critical workplace issues<br>• Self-rated quality of life<br>• Self-rated resilience<br>at post-treatment and at follow-up, respectively	Change from baseline of<br>• Occupational Depression Inventory (ODI)<br>• Beck Depression Inventory-II (BDI-II)<br>• Work Ability Index (WAI)<br>• Return-to-Work Self-efficacy Questionnaire (RTW-SE)<br>• Effort-Reward Imbalance Questionnaire (ERI)<br>• Job Content Questionnaire 2 (JCQ2)<br>• WHOQOL-BREF<br>• Connor-Davidson Resilience Scale (CD-RISC)<br>•<br>•<br>•<br>•<br>at post-treatment and at follow-up, respectively
**Exploratory Endpoints**		To assess the effect of IPT-Work in comparison to SP on<br>• Self-reported days of sick leave throughout the study period	• Number of days of sick leave throughout the study period
**Safety**		To assess the safety of IPT-Work in comparison to SP	Adverse events, including serious adverse events

For all efficacy endpoints, the hypothesis is that IPT-Work is superior to SP. The null hypothesis is equal efficacy.

The primary outcome scale is the clinician-rated 24-item Hamilton Rating Scale for Depression (HRSD-24[21]. The HAM-D/HRSD is the most frequently used measure in depression research and covers the most domains relevant to patients with depression in comparison to other outcome measure[22]. Recent investigations show that the HRSD is a valid and sensitive clinimetric index when conducted by trained raters using a structured form[23]. Higher values of the HRSD-24 correspond to greater severity of depressive symptoms. There are no subscales. Response is defined as a reduction of the HRSD-24 score by at least 50% from baseline. Remission is defined as an HRSD-24 score of < 9.

The following secondary efficacy outcome scales will be used:

- Occupational Depression Inventory (ODI): The ODI is a self-rated questionnaire to assess work-attributed depressive symptoms[24]. Higher scores correspond to greater severity.
- Beck Depression Inventory-II (BDI-II): The BDI-II is a self-rated questionnaire to assess depression severity[25]. Higher scores correspond to greater severity.
- Work Ability Index (WAI): The WAI is a self-rated questionnaire to assess work ability[26]. Higher scores correspond to greater work ability.
- Return to Work Attitude (RTW-SE): is a self-rated questionnaire to assess return-to-work self-efficacy for employees with mental health problems[27]. Higher scores correspond to higher self-rated return-to-work self-efficacy.
- Effort-Reward-Imbalance (ERI): The ERI is a self-rated questionnaire to assess imbalances between work-related efforts and rewards[28]. Higher scores correspond to a higher effort-reward-imbalance. There are three subscales assessing effort (ERI1-ERI6), reward (ERI7-ERI16) and over-commitment (OC1-OC6).
- Job Content Questionnaire 2 (JCQ2): The JCQ2 is a self-rated questionnaire designed to measure the "content" of a respondent's work tasks in a general manner which is applicable to all jobs and jobholders[29]. The JCQ2 covers critical workplace issues that are often overlooked - because they are very difficult to assess - including employee health risks, chronic disease, depression, sickness-absence, disability - and also job satisfaction, active work/creativity, and innovation potential. It is used to measure the high-demand/low-control/low-support model of job strain development and predicts stress-related risk and active-passive behavioral correlates of jobs according to the demand/control model. Higher scores correspond to higher job strain.
- Quality of Life (WHOQOL-BREF): The WHO Quality of Life Instrument (WHOQOL-BREF) is a short form tool consisting of 26 items divided into four domains (physical health, psychological health, social relationships and the environment) to measure quality of life[30–32]. Higher scores correspond to higher domain-specific quality of life.
- Connor-Davidson Resilience Scale (CD-RISC): The CD-RISC is a self-rated questionnaire to assess resilience[30,31]. Higher scores correspond to greater resilience.

In addition, exploratory endpoints include

- Number of self-reported days of sick leave throughout the study period.

Safety is assessed in terms of rates of AEs and SAEs.

2.1 Definition of the primary estimand and full analysis set

The primary estimand (No. 1) corresponds to the primary trial objective (to assess the efficacy of IPT-Work in comparison to SP on depression 3 months after end of treatment) and is based on the primary endpoint (the relative change in HRSD-24 score from baseline to follow-up 3 months after end of treatment (100 – (100 x post/pre) %). It is specified by the following 5 attributes.

- Population: Depressive patients as described by the eligibility criteria, represented by all randomised patients, who will be called full analysis set (FAS)
- Treatments: The randomised experimental arm IPT-Work is compared to the randomised control arm SP regardless of treatment interruptions, treatment discontinuation and prohibited concomitant treatments. Treatment groups are called IPT-Work and SP.
- Variable (endpoint): Relative change in HRSD-24 score from baseline to follow-up
- Intercurrent events: No further intercurrent events other than those specified for the treatments will be considered. They will be addressed by the treatment policy strategy according to ICH E9 (R1) Addendum on Estimands and Sensitivity Analysis in Clinical Trials (ICH, 2019[33]).
- Population level summary: Difference of means (IPT-Work - SP)

2.2 Definition of secondary estimands

2.2.1 Secondary estimands based on the Hamilton Rating Scale for Depression

The following secondary estimands are based on the HRSD-24 scale with the same attributes as the primary estimand regarding population (FAS), treatment (IPT-Work vs SP) and intercurrent events (none other than those specified for the treatments).

Table 2: Variables

No.	Variable (endpoint)	Population level summary
2	Relative change in HRSD-24 score from baseline to post-treatment	difference of means
3	Remission at post-treatment	Odds ratio
4	Remission at follow-up	Odds ratio
5	Response at post-treatment	Odds ratio
6	Response at follow-up	Odds ratio

2.2.2 Further secondary estimands

Further secondary estimands will be based on the change from baseline of

- Occupational Depression Inventory (ODI)
- Beck Depression Inventory-II (BDI-II)
- Work Ability Index (WAI)
- Return to Work Attitude (RTW-SE)
- Effort-Reward-Imbalance (ERI)
- Job Content Questionnaire 2 (JCQ2)
- Quality of Life (WHOQOL-BREF)
- Connor-Davidson Resilience Scale (CD-RISC)

at post-treatment and at follow-up, respectively.

These estimands are defined with the same attributes as the primary estimand regarding population (FAS), treatment (IPT-Work vs SP) and intercurrent events (all ignored). Summary

measures and, where necessary, more detailed definitions of variables (endpoints) will be specified in the SAP.

Safety will be evaluated descriptively.

2.3 Definition of the safety set

The safety set (SAF) includes all randomised patients who have started either IPT-Work or SP group sessions. Treatment cross-overs are impossible to occur due to the logistic arrangements in the trial. Patients are analysed according to the received treatment.

2.4 Estimands: Overview

The following summary and Table 3 give an overview about the definition of the estimands.

Populations:

- FAS, all randomised patients
- SAF, patients who start randomised intervention (treatment cross-overs are impossible to occur due to the logistic arrangements in the trial)

Treatment definition:

- IPT-Work vs SP, as randomised
- AtIPT-Work vs AtSP: as treated, intervention which was started

Intercurrent events:

- Treatment interruptions, treatment discontinuation, prohibited concomitant treatments, non-start (randomised treatment not started)

Table 3: Overview of Estimands

No.	Variable (endpoint) / summary measure	Treatment	Population	Intercurrent events
1	Relative change in HRSD-24 score from baseline to follow-up / difference of means	IPT-Work vs SP	FAS	All ignored
2	Relative change in HRSD-24 score from baseline to end of treatment / difference of means	IPT-Work vs SP	FAS	All ignored
3	Remission at end of treatment / odds ratio	IPT-Work vs SP	FAS	All ignored
4	Remission at follow-up / odds ratio	IPT-Work vs SP	FAS	All ignored
5	Response at end of treatment / odds ratio	IPT-Work vs SP	FAS	All ignored
6	Response at follow-up / odds ratio	IPT-Work vs SP	FAS	All ignored
	Further secondary efficacy endpoints / summary measures to be specified in SAP	IPT-Work vs SP	FAS	All ignored
	(Serious) adverse events / risk difference	AtIPT-Work vs AtSP	SAF	Non-start

3 Study Plan

3.1 Study Design

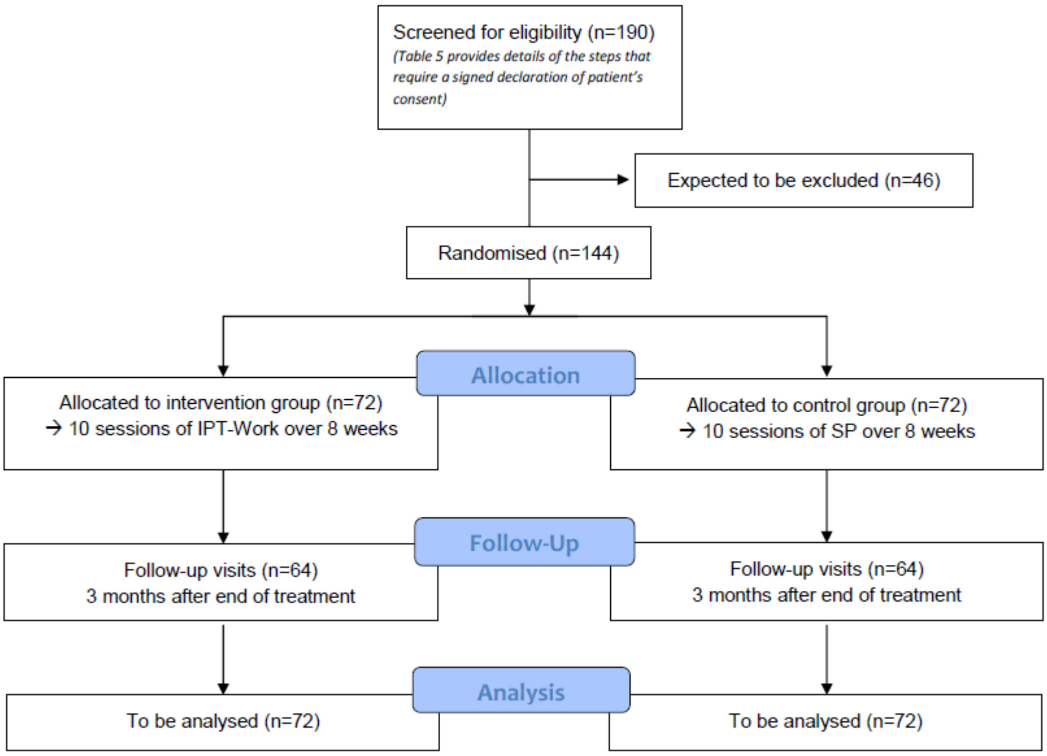

Figure 1: Study Design

Table 4: Timetable for the Study

Informed Consent of first patient (FPFV)	1st quarter 2025
Randomisation of last patient	4th quarter 2026
End of trial for last patient (LPLV)	1st quarter 2027
Statistical analysis	2nd quarter 2027
Treatment duration per patient	2 months

3.2 Participating Centres

6 centres in Germany (2x Freiburg, Mannheim, Frankfurt am Main, Rosenheim/Prien and Hannover), which meet the structural and personnel requirements for performing the planned regular study-related investigations, have been selected and initiated to carry out the trial:

- Department of Psychiatry and Psychotherapy, Medical Center - University of Freiburg (PI: Prof Dr Elisabeth Schramm)
- Department of Psychosomatics, Medical Center - University of Freiburg (PI: Prof Dr Claas Lahmann)
- Department of Psychiatry and Psychotherapy, Central Institute of Mental Health, Medical Faculty Mannheim, University of Heidelberg (PI: Prof Dr Michael Deuschle)
- Department of Psychiatry, Social Psychiatry and Psychotherapy, Hannover Medical School (PI: Prof Dr Kai Kahl)

- Department of Psychiatry, Psychosomatic Medicine and Psychotherapy, University Hospital Frankfurt – Goethe University Frankfurt (PI: Prof Dr Andreas Reif)
- Schoen Clinic Roseneck, Prien am Chiemsee (PI: Prof Dr Dr Andreas Hillert)

Qualifications were evaluated using self-report questionnaires, including patient numbers from previous years, and were checked by a monitor. If necessary, additional qualified centres can be included in the course of the study.

3.3 Number of Study Participants

A target sample size of 144 patients will be enrolled in the study, about 72 in each of the two trial arms. Therefore, about 190 patients will be assessed for eligibility.

3.4 Methods against bias

For logistical reasons, clusters of 4-6 eligible patients at a time will be randomised to group psychotherapy according to the IPT-Work or SP condition. To avoid selection bias, the raters who identify eligible patients and obtain informed consent will not be informed about the randomisation result, nor about ongoing or previous IPT-Work or SP psychotherapy groups at their respective centre.

To further avoid selection bias, the randomisation code to allocate patient groups to interventions will be generated by the CTU using the following procedure to ensure that treatment assignment is unbiased and concealed from patients and site staff. Randomisation of patient groups will be performed, stratified by centre, in blocks of variable length with a 1:1 ratio. The block lengths will be documented separately and will not be disclosed to the centres. The randomisation code will be produced by validated programmes based on the Statistical Analysis System (SAS).

Obviously, blinding of patients and study psychotherapists performing the interventions is not possible. However, data acquisition other than patient reported outcomes will be done by trained blinded clinical raters not involved in patient management. Each site will implement procedures to mask patient treatment assignment from the clinical rater by reminding the patients at each visit not to mention anything that might reveal their treatment condition. Diagnostic training of raters in the SCID-5, HRSD-24 and (S)AE assessments will be conducted by expert trainers and interrater reliability will be ensured. The other measures are self-rated in a standardised questionnaire format.

All confirmatory analyses are fully specified in the trial protocol to avoid reporting bias. The trial is registered prospectively at the German Clinical Trials Register (see chapter 13), and its results will be published irrespective of the outcome to prevent publication bias.

3.5 Roles and Responsibilities

3.5.1 Coordinating investigator

Elisabeth Schramm as the coordinating investigator (CI) is the lead researcher responsible for the overall conduct of this study. The CI oversees the trial's design, implementation, and adherence to ethical and regulatory standards. She facilitates communication among the study sites, manages the data collection process, and ensures the study progresses according to the protocol. The CI also serves as the primary point of contact for regulatory bodies and sponsors.

3.5.2 Principal investigators at the sites

Principal Investigators (PIs) at the sites are the lead researchers at each participating location. They are responsible for the implementation of the study protocol at their respective sites, including the recruitment and management of participants, ensuring compliance with the study's procedures, and overseeing data collection. The PIs communicate with the Coordinating Investigator to report on progress and any issues that arise during the trial.

3.5.3 Rater

Raters evaluate participants' mental health status, symptoms, and progress using standardised instruments. All raters have at least a B.Sc. in Psychology (or comparable) and are trained in a 2-day training to assess study participants based on predefined criteria and measurement tools. They are crucial for maintaining the reliability and validity of the outcomes by providing unbiased assessments. Therefore, raters will remain blind to the participants' group assignments whenever possible to reduce bias in their evaluations (for details see 5.4.1). To ensure adherence and interrater reliability, raters will independently rate videos of HRSD-24 goldstandard ratings (primary outcome) during the training and the study duration.

3.5.4 Psychotherapists

Study psychotherapists are in a completed or far advanced stage of psychotherapy training. All therapists will execute IPT-Work as well as SP groups after thorough training to ensure a high treatment quality (2-day training course in presence in each IPT-Work and SP followed by 3 half-day online booster workshops after the start of the group therapies app. every 6 months). All trainings are led by clinical experts in the field (Elisabeth Schramm, Nicola Thiel, Nadine Zehender, Hannah Piosczyk, John Markowitz or others delegated by the CI). Study therapists will be intensely instructed and trained to adhere to the manual of each condition and not to mix the intervention types. To check for adherence and to support the supervision, a 'Therapeutic Element Checklist' (Stundenbogen) for IPT-Work (e.g. strategies for addressing work-life-balance, demand-control-balance, effort-reward-balance, and work as a social role) and SP (e.g. reflective listening, facilitation of affect, helping the patient to feel understood) is filled out by the therapist immediately after each group session. Separate supervisors (Elisabeth Schramm, Nicola Thiel or others delegated by the CI) for each treatment arm will review the 'Therapeutic Element Checklist' regularly in ongoing supervision. Every second or third session will be supervised by the responsible supervisors (who also conducted the IPT-Work and SP trainings, respectively) in biweekly video conference meetings. Furthermore, a diagnostic training of raters in SCID-5 and HRSD-24 will be conducted in presence (Elisa Scheller, Moritz Elsaeßer, Hannah Piosczyk or others delegated by the CI), and interrater reliability will be ensured.

4 Subject Population and Selection Criteria

According to the recruitment strategy individuals working in firms, institutions (such as clinics, schools, universities), also from outpatient centres and private practices (primary care, psychosomatics, or psychiatry), and media recruited participants will be enrolled in the IPT study. In order to fulfil the recruitment rates within the planned timeframe, sites will be provided with the best possible support finding and recruiting patients into the trial. The recruitment procedure for this randomized controlled trial (RCT) is based on the successful procedure of our pilot study[14]. There, the recruitment process proved to be effective, even under more demanding conditions. Specifically, the pilot study required a larger sample size per centre (n=28), and participants in both groups had to be randomized simultaneously. Despite these challenges, we were able to recruit participants within the planned timeframe. This experience underscores the feasibility and

robustness of our recruitment strategy, which has been slightly refined for the current RCT to further enhance its efficiency.In current practice, respective patients are often not evaluated for depression. Hence, a maximum effort is put to exposure of the targeted patient group to the study sites. To do so, a study website is created to provide checklists, newsletters, and flyers, information about patient informed consent aspects, and letters for resident physicians, etc.

As the general public often regards psychotherapy with or without medication as the preferred treatment option, we will provide corresponding information for treating physicians.

4.1 Inclusion Criteria

Patients eligible for inclusion in this trial must meet **all** of the following criteria:

1. Patient's written informed consent has been obtained
2. Primary diagnosis of Major Depression/MD (single-episode or recurrent) according to the Structured Clinical Interview for DSM-5 (SCID-5-CV; also ICD-10 coded - assessment not older than 14 days on the day of randomisation request)
3. A score of ≥ 17 on the 24-item version of the Hamilton Rating Scale for Depression (HRSD-24) - assessment not older than 14 days on the day of randomisation request
4. A total score of at least 15 on the Occupational Depression Inventory (ODI) - assessment not older than 14 days on the day of randomisation request
5. Sick leave related to depressive or burn-out complaints for at least 7 days during the last 12 months
6. Sufficient German language skills
7. Outpatient status
8. 18-years or older
9. Patients must have a regular work activity that is expected to continue for at least 6 months at the time of randomisation.

4.2 Exclusion Criteria

Patients eligible for this trial must **not** meet any of the following criteria:

1. Acute risk of suicide
2. History of psychotic symptoms, bipolar disorder, or organic brain disorders
3. A primary diagnosis of another SCID-5-CV disorder
4. Concurrent diagnosis of substance dependency
5. Antisocial, schizotypal, or borderline personality disorder (SCID-5-PD)
6. Other ongoing psychotherapy
7. Antidepressive pharmacotherapy (if not stable for at least the last 4 weeks before randomisation)
8. Serious medical condition or time restrictions interfering with participation in regular sessions
9. Current sick leave > 4 weeks
10. Applying for rehabilitation or early retirement
11. Patient without legal capacity who is unable to understand the nature, significance and consequences of the study
12. Simultaneous participation in other studies which could interfere with this study and/or participation before the end of a required restriction period. No prior randomisation in this IPT-Work trial.

13. Persons who are in a disciplinary employment relationship with a member of the study team

5 Procedures

The study procedures for all patients comprise the stages of screening (see 5.1), consent (see 12.1), randomisation (see 5.2), treatment (see 5.3.2 for intervention arm, 5.3.3 for control arm, 5.3.1 for both arms) and follow-up (see 5.4), which are described in detail below. The visit schedule (Table 5) provides a compact overview of the processes for each patient.

Table 5: Visit Schedule

**Trial Period**	**Baseline**		**Treatment**						**Post-treatment**	**Follow-up/ End of Trial**
	**Screening 14 days**	**Randomi-sation**	**8 weeks**						**13 weeks**	
**Visits**	V0	V1	V2	V3	V4	V5	V6-V10	V11	V12	V13
**Week**		W0	W1		W2		W3-7	W8	W9	W21
**Time Window**	day -14 - day 0	day 0	± 4 days	± 4 days	± 7 days	± 7 days	± 7 days	± 7 days	± 7 days	± 7 days
Informed Consent	x									
Inclusion/Exclusion Criteria	x									
Demographics Medical History	x									
Work Context	x								x	x
Structured Clinical Interview for DSM-5 (SCID-5)**	x									
Hamilton Rating Scale for Depression (HRSD-24)	x								x	x
Occupational Depression Inventory (ODI)*	x								x	x
Days of sick leave*	x								x	x
Confirmation of SCID-5, HRSD-24, ODI (if older than 14 days: re-assessment)		x								
Beck Depression Inventory II (BDI-II)*	x								x	x
Work Ability Index (WAI)*	x								x	x
Return to Work Attitude (RTW-SE)*	x								x	x
Effort-Reward-Imbalance (ERI) at work*	x								x	x
Job Content Questionnaire 2 (JCQ2)*	x								x	x
WHOQOL-BREF (Quality of Life)*	x								x	x
Connor-Davidson Resilience Scale (CD-RISC)*	x								x	x
Patient treatment preference*	x								x	
Randomisation**		x								
Individual preliminary talk (see 5.3.1)**			x							
Treatment IPT-Work vs SP sessions**			x	x	x	x	x	x		
Therapeutic element checklist (Stundenbogen)**			x	x	x	x	x	x		
Change of medication/therapy**									x	x
Adverse Events / Serious Adverse Events**			x	x	x	x	x	x	x	x

*Self-rated by patient ** After informed consent

5.1 Patient screening for eligibility

If a patient appears to be eligible for the trial (see 4.1 and 4.2 for inclusion and exclusion criteria), a rater will inform the patient about the trial and ask the patient for his/her written consent, and will assess the patient's eligibility. It is imperative that written consent is obtained prior to SCID-5, which is a trial-specific procedure. The rater will inform the patient that a group of 4-6 eligible patients has to be collected before the entire group will be randomised to the IPT-Work or SP condition and, if necessary, will check the eligibility criteria once more directly before randomisation.

The rater will then record the details of these trial patients on the following trial-specific lists:

- **Subject screening log**: for the documentation of trial patients who were checked for eligibility before and/or during the clinical trial. The following will be entered:
    - consecutive screening number (1, 2, 3 etc.),
    - date of written consent (if obtained),
    - details on whether the patient was randomised,
    - if not randomised, the reason.
    - For all patients who signed informed consent, the individual patient identification code (see below) will be recorded on the subject screening log.
- **Subject identification log**: A confidential log of
    - the names of all trial patients
    - the patient identification code assigned to each patient (see below).

  With this list, the identity of each patient can be revealed. The list must be kept confidential and remain at the trial site. It must not be copied or otherwise be passed on! However, sponsor representatives, clinical research associates (CRAs), auditors and representatives of authorities must be allowed to inspect the list on request.

- **Patient identification code**: A unique trial-specific identification number which identifies the patient and consists of two parts: The first three digits correspond to the site number, the next one or two digits stand for the number of the patient according to the Subject screening log. For example 987-2 (Site No. 987, patient No. 2), so that each patient with consent is numbered uniquely across the entire database.

All patients who have signed the informed consent form will be entered into the trial database.

Patients who have signed the informed consent form but are not randomised between treatments are called screening failures. The reason(s) for screening failure will be documented in the trial database, and their numbers will be given in the respective box in the CONSORT diagram.

For these patients (screening failures), at least the following data will be recorded:

- Date of informed consent
- eCRF pages on fulfilment of eligibility criteria
- Demography data, Medical history, Work context, data about sick leave
- SCID-5, ODI and HRSD-24
- Patient will be randomised between IPT-Work and SP. If a patient is not randomised, a reason (eligibility criteria which are not fulfilled, wish of patient, other: specify) is given.

All randomised patients and their treatment and follow-up data will be documented in the trial database. Further details see Chapter 8.

5.2 Patient randomisation

In this trial groups of 4-6 eligible patients at a time will be cluster-randomised to the IPT-Work or SP condition. When a group of patients is ready for randomisation, the rater will check that SCID-5, HRSD-24 and ODI are not older than 14 days at the day of randomisation request and that all data required for randomisation (see 5.1) are available in the eCRF.

The other data for V1 according to the Visit Schedule (see page 28) must be recorded on paper before randomisation. They should be entered to the trial database within 14 days from randomisation.

After data entry for 4-6 patients, the rater hands over the completed Randomisation Request Form to the study psychotherapist who checks the request, signs and forwards the request to the CTU Freiburg (for contact details see chapter Responsibilities) for randomisation. Forwarding the "Randomisation Request Form" occurs either per FAX or e-mail upon availability at the site. The Randomisation Team at the CTU Freiburg processes the request and returns the randomisation result to the designated persons at the site using FAX or e-mail. The study psychotherapist enters the results into the eCRF.

To maintain the blinding, access to the randomisation module in the eCRF is restricted to the sites study therapist. The same must be ensured for the e-mail or fax reply confirming the randomisation results.

While the blank "Randomisation Request Forms" are filed in the ISF, the returned completed "Randomisation Request Forms" have to be kept at the site in the "Randomisierungs- und Therapiegruppenordner" in a secured place.

The Randomisation Team at the CTU Freiburg processes the Randomisation Request according to the CTU instructions. The Data Management at the CTU confirms the entry of the results. The completed "Randomisation Request Form" is kept at the CTU in a secured place.

Information about each randomisation (without the assignment to one of the arms) is automatically communicated to the Coordinating Investigator, the Principal Investigator at the site concerned and the central Data Management at the CTU. The study psychotherapists will not disclose the randomisation result, nor any information on ongoing or previous IPT-Work or SP psychotherapy groups at his/her centre to the raters involved in patient recruitment and follow-up. The team at the centre keep their personnel who enters data which might reveal the patients' treatment allocation under strict duty of confidentiality.

5.3 Treatment

Based on the result of the randomisation, patients are allocated to one of the two treatment arms. The study intervention (IPT) and the control arm are described in detail below.

5.3.1 General procedures in both study arms

Before the start of the group, in both study arms one individual preliminary talk of app. 30 minutes will be conducted in which an interpersonal inventory is performed and the indication of involvement of the employer and/or a social worker is determined (no documentation in eCRF required).

To check for adherence and to support the supervision, a 'Therapeutic Element Checklist' (Stundenbogen) for IPT-Work (e.g. strategies for addressing work-life-balance, demand-control-balance, effort-reward-balance, and work as a social role) and SP (e.g. reflective listening,

facilitation of affect, helping the patient to feel understood) is filled out by the therapist immediately after each group session.

No additional psychotherapeutic treatment is allowed throughout the study including the follow-up visit.

Antidepressive medication is allowed if stable for at least 4 weeks before randomisation and throughout the follow-up period. The continuous intake of benzodiazepine is prohibited; the selective use of benzodiazepine as rescue medication on-demand for a maximum of 2 weeks is permitted. Other required medication for comorbid somatic diseases will be allowed and documented.

During group sessions, study psychotherapists will encourage patients to report any adverse events either during the session or confidentially. The psychotherapist will document adverse events as described in section 7.

5.3.2 IPT-Work (intervention arm)

The IPT-Work condition follows a manual[34] and focuses on the work context by adding specific elements to the regular IPT strategies in four modules of

1. work-life-balance: identifying work-related stress factors and allostatic overload; psychoeducation on the association of work stress, social support and depression; creating a balance between performance values and interpersonal values; teaching mindfulness skills (as a different therapeutic intervention) to reduce physical and mental tension/stress,
2. demand-control-balance: enhancing communication skills at work (e.g. negotiating modified work tasks or working hours) to cope with difficult work situations; set limits to exaggerated demands (prevent allostatic overload; get a sense of control over the work conditions e.g. organisational injustice); ask for support; cope with interpersonal conflicts and difficult role transitions at the workplace,
3. effort-reward-balance: identify values at work; set self-exertion and reward in balance,
4. work as a “social role”: define your work place with strengths and limitations. The intervention includes 10 group sessions (twice weekly in the first 2 weeks, 1 weekly thereafter) of 90 minutes for 4-6 outpatients over 8 weeks.

5.3.3 Supportive Psychotherapy (SP, control arm)

SP is a manual-based[16], nonspecific, non-work-directed psychotherapeutic intervention found to be effective in the treatment of depression[17]. SP will be applied in the same format (group sessions), frequency, intensity, and duration as the IPT-Work condition in order to implement a homogeneous and comparable control group. It resembles supportive clinical management or client-centred counseling and includes psychoeducational elements and other common aspects of psychotherapy, such as reflective listening, facilitation of affect, helping the patient to feel understood, empathy, hope, and therapeutic optimism. Specific interpersonal, cognitive, behavioral, and psychodynamic interventions were explicitly proscribed. Since SP is based on exclusively common therapy effects (common factors approach) and IPT-Work elicits common as well as specific therapy effects, using SP as control group enables an estimate of the specific effects of IPT-Work beyond the common therapy effects.

5.4 Follow-up evaluations

5.4.1 Blinding of raters

At the beginning of each interview, patients are reminded that the raters are blinded. They are therefore asked to refrain from making any references in the interview that would allow the rater to draw conclusions about the study arm that the patient has been assigned to.

If a rater for any reason is unintentionally unblinded, this does not constitute a reason for the patient to be excluded from the study. Depending on the personnel resources of the local study team, the unblinded rater should be replaced by another, blinded rater. If this is not possible, data that is collected unblinded must be labelled as such.

5.4.2 Post-treatment, Follow-up / End of Trial

After end of treatment, patients will participate in two subsequent evaluations at post-treatment (W9 ± 7 days) and follow-up (W21 ± 7 days) to assess their condition after study psychotherapy. All evaluations will be conducted by the respective trained and blinded raters (preferably the same person who was responsible for the study inclusion) according to the details described above.

6 Discontinuation criteria

6.1 Premature termination of one of the treatment arms or the entire study

The coordinating investigator is under obligation to monitor the progress of the study with regard to safety-relevant developments and, if necessary, initiate the premature termination of a treatment arm or the entire study. The coordinating investigator will be supported in this responsibility by the DSMB, if necessary.

A study arm or the entire study must be terminated prematurely if:

- the benefit-to-risk ratio for the patients changes markedly,
- the coordinating investigator or the DSMB considers that the termination of the study is necessary,
- indications arise that the study patients' safety is no longer guaranteed,
- the questions(s) addressed in the study can be clearly answered on the basis of results of another study on the same subjects,
- an insufficient recruitment rate makes a successful conclusion of the study unrealisable or no longer feasible.

If the study is prematurely terminated or suspended for any reason, the investigator should promptly inform the study patients and ensure appropriate therapy and follow-up for the patients. Where required by the applicable regulatory requirements, the IEC(s) will also be informed (this is usually done by the coordinating investigator).

6.2 Premature termination of the study at one of the sites

The investigator or coordinating investigator has the right to terminate the study at one of the sites.

The study can be terminated prematurely at a site by the investigator if, for instance unforeseeable circumstances have arisen at the site which preclude the continuation of the study, the investigator considers that the resources for continuation are no longer available, the investigator considers that the continuation of the study is no longer ethically or medically/psychologically justifiable.

The coordinating investigator can initiate the exclusion of a site from further participation if, for instance, patient recruitment is inadequate, serious problems arise with regard to the quality of the collected data which cannot be resolved.

Premature termination at one of the sites does not automatically mean a termination of the study for already enrolled patients. A separate decision on further treatment must be made for each patient, depending on the overall situation. Adequate further treatment and follow-up of already enrolled patients must be ensured. The documentation of already enrolled patients will be reviewed for completeness and plausibility. Queries may be raised for further clarification before the site is closed. These queries must be answered properly by the site. The IEC(s) must be duly notified of the site´s closure, including reasons, within the specified period. The study site concerned will be closed in stages by the CRA when a decision has been made on the further treatment of the patients concerned.

6.3 Discontinuation of study treatment or study participation for individual patients

It has to be distinguished if study treatment of a patient has been stopped prematurely or if the study participation of a patient was stopped prematurely.

In the case study treatment of a patient has been stopped prematurely, further follow-up visits and the assessment of the study endpoints are essential to enable an analysis of the full analysis set according to the intention-to-treat principle. Further visits, follow-up and documentation should always be striven for / ensured in this case. This includes the follow-up of AEs, the time of termination, the results available at that time and, if known, the documentation of the termination of treatment on the eCRF and in the medical/psychological record, giving reasons, a final examination and documentation according to the protocol (if possible).

In the case study participation of a patient was stopped prematurely, the conduct of further follow-up visits is no longer possible. The documentation should be completed as far as possible under these circumstances, e.g. a final examination and documentation according to the protocol (if possible), a documentation of the premature study termination on the eCRF and in the medical record, giving reasons, appropriate further treatment and follow-up outside the study should be ensured.

A study patient can withdraw his/her consent at any time, without having to give reasons, and have his/her entire study participation terminated prematurely. However, the prerequisite for this is that the patient actively terminates study participation by withdrawing his/her consent for the follow-up and documentation.

The responsible investigator may only withdraw a patient from participation in the study in case of extreme circumstances arise which make any study-relevant follow-up impossible.

7 Safety monitoring and documentation

7.1 Definition of adverse events (AEs) and serious adverse events (SAEs)

An AE in this study is defined as any untoward medical occurrence in a patient who has been randomised.

The following adverse events (AEs) will be documented to monitor safety of randomised patients according to one of the following categories (select main category)

A) Worsening of psychopathological symptoms

B) Appearance of new psychopathological symptoms

C) Passive suicidal thoughts

D) Active suicidal thoughts

E) AEs related to the patient–therapist relationship

F) AEs related to personal life

G) AEs related to occupational life

H) Other (i.e., non-psychopathological) AEs (e.g. influenza or fractures)

A serious AE (SAE) in this study is defined as any untoward medical occurrence that results in any of the following outcomes:

- Death
- Life-threatening situation (patient is at immediate risk of death)
- Inpatient hospitalisation or prolongation of existing hospitalisation (pre-planned hospitalisations do not have to be considered)
- Persistent or significant disability/incapacity
- Failed suicide attempt
- Other important medical event: An event that does not fit any of the other outcomes above, but may jeopardise the patient and may require medical or surgical intervention (treatment) to prevent one of the other outcomes above.

These definitions are based on a standardised questionnaire already applied in previous psychotherapy trials[18–20,35].

7.2 Documentation of AEs and serious AEs (SAEs)

(Serious) adverse events have to be documented in the eCRF beginning from the date of randomisation and ending with the date of the study end.

The documentation obligation of AEs applies to the study psychotherapist or rater who first became aware of the event.

- Characterisation of the event (see above listed events, in case of “Other AEs”, a diagnosis/symptoms have to be provided, symptoms only if diagnosis not yet available)
- Onset/end date
- Severity (mild to severe)
- Relationship to study procedure
- Serious / non-serious (yes/no; yes, if one of the SAE criteria is met)
- Outcome

7.3 SAE reporting

In case an adverse event meets the seriousness criteria (see section 7.1) the SAE-Reporting-Form, as filed in the ISF, has to be filled out. Details about the process are specified on this form.

7.4 Suicidality

Suicidality will be assessed at the screening visit and at every following visit via clinical interview conducted by the rater and during the assessment of serious adverse events and adverse events. In addition, cut-off thresholds in the HRSD-24 (Item 3 > 1) and BDI-II (Item 9 > 1) are checked. In case of suicidality, the rater has to determine the risk level: At low risk, the patient has passive suicidal ideations, but no suicidal intentions, plans or active wish to commit suicide. All study personnel should pay attention to suicidality and assess the change of status carefully at every

visit. The patient must be informed about help services and emergency plans in case of worsening of symptoms.

At increased risk, the patient has suicidal intentions or plans, but no active wish to commit suicide. This is an urgent situation. The study personnel have to ensure that the patient is being seen by a psychiatrist within 24 hours. The patient must be informed about help services and emergency plans in case of further worsening of symptoms.

At acute risk, the patient has active suicidal intentions or plans. Self-control or external social support might be lacking (e.g. family, friends) or be unclear. The study personnel must immediately initiate the admission of the patient to a psychiatric inpatient unit.

8 Data Collection and Management

8.1 Data confidentiality

Information about trial patients will be kept confidential and managed under the applicable laws and regulations. Those regulations require a signed patient authorisation informing the patient of the following:

- which protected health information (PHI) will be collected from patients in this trial;
- who will have access to that information and why;
- who will use or disclose that information;
- the rights of a research patient to revoke their authorisation for use of their PHI.

In the event that a patient revokes authorisation to collect or use PHI, the investigator, by regulation, retains the ability to use all information collected prior to the revocation of patient authorisation. For patients that have revoked authorisation to collect or use PHI, attempts should be made to obtain permission to collect at least vital status (i.e. that the patient is alive) at the end of their scheduled trial phase.

The data collection system for this trial uses built-in security features to encrypt all data for transmission in both directions, preventing unauthorised access to confidential participant information. Access to the system will be controlled by individually assigned user identification codes and passwords, made available only to authorised personnel who have completed prerequisite training.

8.2 Documentation of trial data

8.2.1 Documentation in medical records

The investigator will record the participation in the trial, the frequency of the trial visits, the relevant medical data, the concomitant treatment and the occurrence of adverse events in the medical record of each trial patient.

8.2.2 Documentation in (e)CRF

An electronic data capture (EDC) system will be used in this trial. All data collected during the trial will be entered on the trial-specific e-forms by the responsible investigator, or designated person, as timely as possible. Data entry and data corrections on e-forms are automatically tracked in the audit trail created by the EDC system. The investigator, or a deputy who is designated by the investigator, will document the trial data in the eCRF as promptly as possible, but not later than two weeks after patient´s visit.

Data corrections in the e-CRF due to queries are performed by the responsible investigator, or designated person, as timely as possible.

8.3 Data management

The data management will be performed with REDCap™, a fully web based remote data entry system based on web forms, which is developed and maintained by the REDCap™ Consortium (https://projectredcap.org/about/consortium/).

The technical specifications of the database will be described in the codebook delivered automatically by the REDCap™ system.

Details on data management (software, procedures, responsibilities, etc.) will be described in a data management manual prior to the trial. During the trial, the performance of data management and any deviations from the data management manual will be documented. Technical specifications of the trial data base and all data checks will be documented in the Study Data Dictionary and Codebook.

The trial data base has been fully validated before any data entry will be performed. Data entry personnel will not be given access to the trial data base until they have been trained. An audit trail provide a data history which data were entered, changed or deleted, by whom and when.

Data will be checked during data entry by so-called edit checks. If applicable, the data will be reviewed for completeness, consistency, plausibility, and regarding protocol violations and other distinctive problems (e.g. cumulative missings) using SAS software. The resulting queries will be sent to the investigator at the site for correction or verification of the documented data. The queries should be completed within 14 days.

All programmes which can be used to influence the data or data quality will be validated (e.g. edit check and data validation programmes, programmes for eCRF/query tracking or for import of external data, etc.).

8.4 Data coding

Concomitant medications entered into the database will be coded using the WHO Drug Reference List. Concomitant therapies will be described based on a study-specific pre-specified list.

Medical history/current medical conditions will not be coded. A study-specific pre-specified list of categories will be used in the eCRF.

Adverse events will be categorised as specified in section 7.1.

9 Quality Assurance System

During the study, quality control and quality assurance will be ensured through monitoring, auditing and supervision by the authorities.

9.1 Quality Control (Monitoring)

The investigators at all trial sites will grant the CRA access to the patients' personal medical records for the verification of the proper documentation of study data. The provisions of the Federal Data Protection Act will be fully observed (the CRA is bound by medical secrecy when comparing the CRFs with the source documents).

All investigators agree that the CRA will visit the centre before, during and after completion of the study. The investigator must allow sufficient time for these visits, alternatively the CRA may be provided with other trained staff for assistance during the visits. The investigator will grant the CRA access to the source documents for the fulfilment of his/her duty. The aim and purpose of these visits is, in particular:

- evaluation of the progress of the study,

- check for compliance with the study protocol,
- CRF review for accuracy and completeness,
- CRF validation against source data,

A monitoring reports will be written on each visit. This will document the progress of the study and give an account of all problems that occurred (e.g. refusal of inspection).

Between visits, the responsible CRA will maintain regular telephone contact with the study centres. Further detailed information on monitoring will be recorded in a monitoring plan.

9.2 Source Data Verification (SDV)

Source data verification will be performed in order to verify the accuracy and completeness of the entries on the case report form (CRF) by comparing them with the source data, and to ensure and increase the quality of the data. All data which are patient to SDV must have been entered in the medical record or, in the case of source documents, enclosed with the medical record. The investigators will afford the CRA access to the medical records for the performance of SDV.

Source data as defined by ICH-GCP include data such as hospital records, clinical and office charts, laboratory notes, memoranda, patients' diaries or evaluation checklists, pharmacy dispensing records, recorded data from automated instruments, copies or transcriptions certified after verification as being accurate copies, microfiches, photographic negatives, microfilm or magnetic media, x-rays, and records kept at the pharmacy, at the laboratories and at medico-technical departments involved in the clinical study.

9.3 Quality Assurance (Auditing)

According to the ICH-GCP guidelines, audits will be performed according to a quality assurance system. These may be conducted by the coordinating investigator, the Medical Center Freiburg or by an authority.

During an audit, the planning, conduct and analysis of a study will be checked for compliance with the national laws and the requirements of the ICH-GCP guidelines.

This includes checking the data management and organisation at the study site and inspecting institutions, laboratories and source documents. The aim of quality assurance is to ensure that the results and conclusions described in the final report can be correctly deduced from the raw data.

All persons who conduct an audit undertake in writing to treat all data which are patient to medical secrecy or could reveal the patient's identity in absolute confidence, and to restrict the use of such data to the purposes agreed by the patient in writing.

Proposed audit dates, characteristics of the selected patients and further information will be transmitted to the investigator by the CRA in a timely manner.

10 Biostatistical Planning and Analysis

Before the start of the final analysis a detailed statistical analysis plan (SAP) will be prepared. If the SAP contains any changes to the analyses outlined in the trial protocol, they will be marked as such, and reasons for amendments will be given.

All statistical programming for analysis will be performed with the Statistical Analysis System (SAS) or R.

10.1 Trial Design

For details on trial design see section 3.1 of the protocol.

10.2 Blinding of study statistician

A member of the CTU who is not involved in the trial will produce programme code to generate randomisation lists providing allocation of patient groups of size 4-6 to the IPT-Work or SP condition. The study statistician will provide the details for the implementation of the randomisation lists, which will be stratified by centre, in blocks of variable length with a 1:1 ratio, in a document which will not be disclosed to the centres.

All analysis programmes regarding efficacy endpoints (see section 2) will be prepared by the study statistician using a dummy treatment variable. No evaluation of efficacy endpoints by randomised treatment will be allowed before the final database lock.

10.3 Definition of estimands and analysis sets

For details on the definition of estimands, analysis sets defined in connection with estimands, and endpoints see section 2.

In addition, the analysis set IC (informed consent) is defined as all patients who give informed consent, and the analysis set SCF (screening failure) is defined as all patients who give informed consent but are not randomised. These analysis sets are defined for descriptive purposes according to the CONSORT statement.

10.4 Sample size calculation

In the pilot trial, mean±SD relative changes (decreases) from baseline to follow-up of HRSD-24 were 62.2%±28.0% and 22.6%±71.6% for IPT-Work and TAU, respectively[14], data not shown). On the basis of a two-group t-test at two-sided significance level of 5%, a sample size of 62 observations per arm yields a power of 80% to detect a difference if the HRSD-24 mean relative changes from baseline at follow-up are assumed to differ by 28.0% points with a common SD of 55.0% (nQuery Version 9.2.0.0). The common SD is estimated to lie in between SDs seen in the pilot trial for IPT-Work and TAU, since IPT-Work and SP have similar treatment intensity. The means difference is considered both minimum clinically relevant[36,37] and realistic since IPT is generally more effective than SP[17] and IPT-Work is specifically tailored to depression in a work context[12,34]. This calculation is conservative given that evaluation in a linear model adjusting for baseline measurement will further increase the power. Due to IPT-Work and SP group sizes (clusters) of only 4-6 participants and the fact that the interventions are directed at patients, the design effect induced by intracluster correlation is conservatively estimated as 1.04[38], yielding an increase to 62x1.04≈64 observations per arm. Given an anticipated drop-out rate at follow-up of 10%, 128/0.9≈144 patients should be randomised.

10.5 Methods of analysis

10.5.1 Descriptive statistics

Continuous data will be summarised by arithmetic mean with 95%-confidence interval (CI), standard deviation, minimum, 25% quantile, median, 75% quantile, maximum, and the number of complete and missing observations if greater than zero. If appropriate, continuous variables can also be presented in categories.

Categorical data will be summarised by the total number of patients in each category and the number of missing values if greater than zero. Relative frequencies are displayed as valid % (100 times number of patients divided by the number of patients with non-missing values). For ordered categorical data, the corresponding cumulative relative frequencies will also be shown.

Percentages of patients with missing values will be displayed where this percentage may be of special interest. This may be particularly relevant where the absolute numbers of patients and/or the absolute numbers of patients with missing values differ between treatment arms.

Questionnaires will be evaluated according to their respective manual. For scores and sub scores descriptive statistics and frequencies will be provided by intervention and time point.

Except for the analysis concerning the primary estimand, all other statistical analyses will be considered as descriptive.

10.5.2 Patient recruitment, disposition of patients

The dates of first and last informed consent, of first and last randomisation, and of the last visit of the last patient will be indicated. A flow chart according to the CONSORT statement will be given. The corresponding descriptive evaluations will be presented in frequency tables.

10.5.3 Patients‘ baseline characteristics

Demographic and other baseline data will be summarised descriptively in total and by treatment arm using the FAS. To capture seasonal effects, this will include the calendar month (January, February,…) in which the group therapy started.

10.5.4 Trial interventions

The number of therapy groups, the frequency of group sizes as randomised (4, 5 or 6), and the frequency of actual group sizes (which can be lower in case a patient does not start group therapy) will be presented in total, by centre and by treatment arm.

The number of attended group sessions per patient will be summarised both as continuous and as categorical variable in total and by treatment arm. The number of patients who attended a minimum number of group sessions, to be specified in the SAP, will be summarised in total and by treatment arm. In addition, the mean number and the mean percentage (out of 4, 5 or 6, or possibly fewer in case a patient does not start group therapy) of patients who attended each specific out of the ten group sessions will be presented in total and by treatment. Patients' treatment preferences at baseline and post-treatment will be evaluated descriptively.

10.5.5 Concomitant treatment and medication

The study team of the coordinating investigator will review and classify therapeutic interventions and concomitant medication as “allowed”/“not allowed” (see 5.3.1) based on corresponding listings, blinded to treatment allocation, to be provided to the study team by the CTU after database lock. The number of patients with any additional psychotherapeutic treatment throughout the study including the follow-up visit, the number of patients who took antidepressive medication or benzodiazepines not allowed during the study, as well as all concomitant medication as a whole will be presented in total and by treatment. The concomitant medications will be summarised by ATC level 3. In each table, patients will be counted once if they took at least one medication from the respective ATC level.

10.5.6 Adherence of study therapists and interrater reliability

The study team of the coordinating investigator will evaluate the adherence of study therapists to the respective manuals for IPT-Work and SP based on the ‘Therapeutic Element Checklist' (Stundenbogen). It will also evaluate interrater reliability with respect to SCID-5 and HRSD-24 observed during diagnostic training of raters. The methods will be specified outside this protocol.

10.5.7 Analysis of primary estimand

Main estimator for estimand No. 1

In the primary analysis, all randomised patients will be analysed as belonging to their randomised arm, regardless of any protocol deviations. The effects of SP and IPT-Work on the primary endpoint (difference of means [IPT-Work minus SP] of the relative change in HRSD-24 score from baseline to follow-up) will be estimated and tested in a linear mixed model for repeated measures (MMRM). The model will include randomised arm (IPT-Work and SP), time point (post-treatment and follow-up), their interaction, continuous HRSD-24 baseline scores and their interaction with time point as fixed independent variables and the therapy group (cluster) as random effect. The model will allow for intrasubject correlation using a compound symmetry correlation structure. The two-sided test at significance level 5% for a difference between IPT-Work and SP at follow-up will be based on the two-sided 95% confidence interval derived for the corresponding difference in least-squares means estimated the linear MMRM. The analysis assumes that missing values are missing at random (MAR), indicating that they are related to the independent variables, but not to the unmeasured outcomes themselves. No efficacy interim analysis will be performed.

Sensitivity estimator for estimand No. 1

If more than 10% of the HRSD-24 score measurements at follow-up are missing in at least one arm, a sensitivity analysis will be performed. In both arms, missing values will be multiply imputed from the SP arm of the primary MMRM. The analysis assumes that missing values are missing not at random (MNAR), and that values missing in the IPT-Work and the SP arms are similar to those observed in the SP arm. The results will be combined using Rubin's rules for multiple imputation[39].

Supplementary analyses

To assess the correlation among scores of patients treated in the same SP or IPT-Work therapy group, respectively, the intracluster correlation (ICC) will be estimated from the MMRM for descriptive purposes.

An exploratory analysis will be conducted to assess the consistency of the treatment effect across centres and genders using point estimates with confidence intervals and interaction terms.

10.5.8 Analysis of secondary estimands

The difference of means between IPT-Work and SP with respect to the relative change in HRSD-24 score from baseline to post-treatment (estimand No. 2) will be derived from the same linear MMRM as the primary estimand.

For the binary outcomes based on the HRSD-24 score (estimands No. 3-6, remission and response), the analysis will be performed in a generalised linear mixed model with a logistic link function, to be specified in the SAP, under the assumption that missing values are missing at random. If more than 10% missing values occur in at least one arm at follow-up, a sensitivity analysis will be performed based on the multiply imputed HRSD-24 scores generated for the primary estimand, which will be used to multiply replace the corresponding missing remission/response status.

For the further secondary estimands, change from baseline (difference post-pre) will be analysed in a linear mixed model for repeated measures to be specified in the SAP.

10.5.9 Analysis of safety

Adverse events are described by main categories A-H (see section 7.1). The incidences of adverse events will be summarised in the SAF by treatment arm with two-sided 95% confidence intervals based on Wilson scores (without continuity correction) according to method 3 in Newcombe (1998)[40].

The AEs will be displayed in summary tables by treatment as follows:

The total number of AEs, the minimum, maximum and mean number of AEs per patient will be calculated per main category A-H and in total.

The incidence of AEs defined by main categories A-H will be calculated as the number of patients who experienced at least one AE with the respective category A-H in percentage of the total number of patients.

Each table will be produced for the following AE-sets:

- All AEs
- All Serious Adverse Events (SAEs)

Additionally, these tables will be recalculated for AEs with onset during the treatment period and for AEs with onset after the treatment period.

11 Data Safety Monitoring Board (DSMB)

An independent Data Safety Monitoring Board (DSMB) was be established before enrolment of the first patient. The members of the DSMB are given in section “Responsibilities”. The function of the DSMB is to monitor the course of the trial and if necessary to give a recommendation to the coordinating investigator for continuation, modification or discontinuation of the trial. The underlying principles for the DSMB are ethical and safety aspects for the patients. It is the task of the DSMB to examine, whether the conduct of the trial is still ethically justifiable, whether safety of the patients is ensured, and whether the process of the trial is acceptable. For this the DSMB has to be informed about the patient recruitment, the adherence to the protocol, and the observed adverse events. The composition and responsibilities of the DSMB, the structure, frequency and procedures of its meetings, and its relationship to other key trial team members, will be laid down in a separate DSMB charter.

12 Ethical and Legal Principles

12.1 Subject Informed Consent

Before enrolment in the study, the subject will be informed that participation is voluntary and that he/she may withdraw at any time without having to give reasons and without penalty or loss of benefits to which the subject is otherwise entitled.

The subject will be given ample time and opportunity to obtain answers to any open questions. All questions should be answered to the satisfaction of the subject. In addition, the subject will be given a "Subject Information Sheet“, which contains all the important information in writing.

The subject's written consent must be obtained before any study-specific tests.<br>For this purpose, the written consent form will be personally dated and signed<br>by the study subject and the rater conducting the informed consent discussion.

By signing the consent form, the subject agrees to voluntarily participate in the study and declares that he/she agrees to be contacted for the follow up visits. By signing the form, the subject also declares that he/she agrees to the recording of personal data, particularly medical data, for the study after pseudonymisation.

After signing, the subject will be given one copy of the signed and dated written consent form and any other written information to be provided to the subjects.

12.2 Ethical and Regulatory Requirements

Before the start of the study (enrolment of the first patient), the principal investigator has submitted the study documents to the responsible Ethics Committee in Freiburg for approval. The personnel and facilities' qualifications of the participating sites are verified by the principal investigator before submission to the local ethics authorities (where applicable according to local federal state law). It is not required to obtain authorisation from a competent authority for IPT Work.

12.3 Data Protection and Confidentiality

The pertinent provisions on data protection must be fully complied with.

The study subjects will be informed of the purpose and extent of the collection and use of personal data, particularly medical data.

Findings obtained in the course of the cohort study will be stored on electronic media and treated in strict confidence. For the protection of these data, organisational measures have been taken to prevent disclosure to unauthorised third parties. For example, the subject data will be captured in pseudonymised form (subject ID No. for the particular study, year of birth) throughout the documentation and evaluation phase.

12.4 Archiving

After completion of the trial the Subject identification log and all essential study documents will be retained at the study sites for 10 years. The local PI will be responsible for the achiving compliant to GCP.

After completion of the trial the TMF will be retained at the CI’s sites for 10 years. The CI will be responsible for the achiving compliant to GCP

13 Registry and Publications

The Coordinating Investigator has posted the key elements of this protocol in DRKS (DRKS ID: DRKS00035259) before the start of the recruitment phase. Additionally the protocol, will be published after initival approval of the ethics committee in a scientific journal. In addition, upon trial completion the results of this trial will be submitted for publication to a peer-reviewed journal.

Reporting guidelines will be taken into account (see www.equator-network.org), e.g. the CONSORT statement should be adhered to in the preparation of papers on the results of randomised studies.

Each publication of trial results will be in mutual agreement between the principal investigator, the other investigators involved, the biostatistician and the CTU. All data collected in connection with the clinical trial will be treated in confidence by the coordinating investigator and all others involved in the trial, until publication. Final results may only be published (orally or in writing) with the agreement of the coordinating investigator, the biostatistician and the CTU. This is indispensable for a full exchange of information between the above-named parties, which will ensure that the opinions of all parties involved have been heard before publication. The agreement, which does

not include any veto right or right of censorship for any of the parties involved, may not be refused without good reason.

14 Administrative agreements

14.1 Financing of the project

The clinical trial is financed by the German Research Foundation (DFG); its project number is 507692077, file reference: SCHR 443/18-1.

This funding source had no role in the design of this study and will not have any role during its execution, analyses, interpretation of the data, or decision to submit results.

14.2 Study reports

Starting in December 2024, reports will be periodically submitted to the German Research Foundation (DFG). The interim reports are prepared in accordance with the specifications of the German Research Foundation (Clinical Trials Programme). The interim reports serve to monitor the progress of the approved study on a six months basis and can be submitted to the review group for renewed discussion if the milestones are not reached. Except when required by law, no one will disclose a result of the study to third parties unless all parties involved have first agreed on the results of the analysis and their interpretation.

15 References

1. Roesler U, Jacobi F, Rau R. Work and mental disorders in a German national representative sample. *Work Stress*. 2006;20(3):234-244. doi:10.1080/02678370601008463

2. Jacobi F, Höfler M, Siegert J, et al. Twelve-month prevalence, comorbidity and correlates of mental disorders in Germany: the Mental Health Module of the German Health Interview and Examination Survey for Adults (DEGS1-MH). *Int J Methods Psychiatr Res*. 2014;23(3):304-319. doi:10.1002/mpr.1439

3. Kessler RC, Merikangas KR, Wang PS. The Prevalence and Correlates of Workplace Depression in the National Comorbidity Survey Replication. *J Occup Environ Med Am Coll Occup Environ Med*. 2008;50(4):381-390. doi:10.1097/JOM.0b013e31816ba9b8

4. Lerner D, Henke RM. What does research tell us about depression, job performance, and work productivity? *J Occup Environ Med*. 2008;50(4):401-410. doi:10.1097/JOM.0b013e31816bae50

5. Angerer P, Gündel H, Kröger C, Rothermund E. Rationale, Modelle und Wirkung arbeitsplatznaher psychotherapeutischer Angebote. *Bundesgesundheitsblatt - Gesundheitsforschung - Gesundheitsschutz*. 2024;67(7):743-750. doi:10.1007/s00103-024-03892-8

6. Psychoreport 2019 - DAK-Gesundheit. Accessed February 5, 2020. https://www.dak.de/dak/download/190725-dak-psychoreport-pdf-2125500.pdf

7. Hansson M, Chotai J, Bodlund O. Patients' beliefs about the cause of their depression. *J Affect Disord*. 2010;124(1):54-59. doi:10.1016/j.jad.2009.10.032

8. Du Prel JB, Koscec Bjelajac A, Franić Z, et al. The Relationship Between Work-Related Stress and Depression: A Scoping Review. *Public Health Rev*. 2024;45:1606968. doi:10.3389/phrs.2024.1606968

9. Modini M, Joyce S, Mykletun A, et al. The mental health benefits of employment: Results of a systematic meta-review. *Australas Psychiatry*. 2016;24(4):331-336. doi:10.1177/1039856215618523

10. Nieuwenhuijsen K, Faber B, Verbeek JH, et al. Interventions to improve return to work in depressed people. *Cochrane Database Syst Rev*. 2014;(12):CD006237. doi:10.1002/14651858.CD006237.pub3

11. Bonde JPE. Psychosocial factors at work and risk of depression: a systematic review of the epidemiological evidence. *Occup Environ Med*. 2008;65(7):438-445. doi:10.1136/oem.2007.038430

12. Schramm E, Berger M. Interpersonelle Psychotherapie bei arbeitsstressbedingten depressiven Erkrankungen. *Nervenarzt*. 2013;84(7):813-822. doi:10.1007/s00115-013-3744-5

13. Cuijpers P, Andersson G, Donker T, Van Straten A. Psychological treatment of depression: Results of a series of meta-analyses. *Nord J Psychiatry*. 2011;65(6):354-364. doi:10.3109/08039488.2011.596570

14. Schramm E, Mack S, Thiel N, Jenkner C, Elsaesser M, Fangmeier T. Interpersonal Psychotherapy vs. Treatment as Usual for Major Depression Related to Work Stress: A Pilot Randomized Controlled Study. *Front Psychiatry*. 2020;11. doi:10.3389/fpsyt.2020.00193

15. Niedermoser DW, Kalak N, Kiyhankhadiv A, et al. Workplace-Related Interpersonal Group Psychotherapy to Improve Life at Work in Individuals With Major Depressive Disorders: A Randomized Interventional Pilot Study. *Front Psychiatry*. 2020;11:168. doi:10.3389/fpsyt.2020.00168

16. Markowitz JC. What is Supportive Psychotherapy? *Focus*. 2014;12(3):285-289. doi:10.1176/appi.focus.12.3.285

17. Cuijpers P, Driessen E, Hollon SD, van Oppen P, Barth J, Andersson G. The efficacy of non-directive supportive therapy for adult depression: A meta-analysis. *Clin Psychol Rev*. 2012;32(4):280-291. doi:10.1016/j.cpr.2012.01.003

18. Schramm E, Kriston L, Zobel I, et al. Effect of Disorder-Specific vs Nonspecific Psychotherapy for Chronic Depression: A Randomized Clinical Trial. *JAMA Psychiatry*. 2017;74(3):233-242. doi:10.1001/jamapsychiatry.2016.3880

19. Dafsari FS, Bewernick B, Biewer M, et al. Cognitive behavioural therapy for the treatment of late life depression: study protocol of a multicentre, randomized, observer-blinded, controlled trial (CBTlate). *BMC Psychiatry*. 2019;19(1):423. doi:10.1186/s12888-019-2412-0

20. Dafsari FS, Bewernick B, Böhringer S, et al. Cognitive Behavioral Therapy for Late-Life Depression (CBTlate): Results of a Multicenter, Randomized, Observer-Blinded, Controlled Trial. *Psychother Psychosom*. 2023;92(3):180-192. doi:10.1159/000529445

21. Hamilton M. A RATING SCALE FOR DEPRESSION. *J Neurol Neurosurg Psychiatry*. 1960;23(1):56-62. doi:10.1136/jnnp.23.1.56

22. Veal C, Tomlinson A, Cipriani A, et al. Heterogeneity of outcome measures in depression trials and the relevance of the content of outcome measures to patients: a systematic review. *Lancet Psychiatry*. 2024;11(4):285-294. doi:10.1016/S2215-0366(23)00438-8

23. Carrozzino D, Patierno C, Fava GA, Guidi J. The Hamilton Rating Scales for Depression: A Critical Review of Clinimetric Properties of Different Versions. *Psychother Psychosom*. 2020;89(3):133-150. doi:10.1159/000506879

24. Bianchi R, Schonfeld IS. The Occupational Depression Inventory: A new tool for clinicians and epidemiologists. *J Psychosom Res*. 2020;138:110249. doi:10.1016/j.jpsychores.2020.110249

25. Beck AT, Steer RA, Brown G. Beck Depression Inventory–II. Published online September 12, 2011. doi:10.1037/t00742-000

26. Tuomi, K., Ilmarinen, J., Jahkola, A., Katajarinne, L. and Tulkki, A. Work Ability Index. 2nd Edition,. Published online 1998.

27. Lagerveld SE, Blonk RWB, Brenninkmeijer V, Schaufeli WB. Return to work among employees with mental health problems: Development and validation of a self-efficacy questionnaire. *Work Stress*. 2010;24(4):359-375. doi:10.1080/02678373.2010.532644

28. Siegrist J, Starke D, Chandola T, et al. The measurement of effort–reward imbalance at work: European comparisons. *Soc Sci Med*. 2004;58(8):1483-1499. doi:10.1016/S0277-9536(03)00351-4

29. Karasek R, Brisson C, Kawakami N, Houtman I, Bongers P, Amick B. The Job Content Questionnaire (JCQ): An instrument for internationally comparative assessments of psychosocial job characteristics. *J Occup Health Psychol*. 1998;3(4):322-355. doi:10.1037/1076-8998.3.4.322

30. Sarubin N, Gutt D, Giegling I, et al. Erste Analyse der psychometrischen Eigenschaften und Struktur der deutschsprachigen 10- und 25-Item Version der Connor-Davidson Resilience Scale (CD-RISC). [First analysis of the 10- and 25-item German version of the Connor-Davidson Resilience Scale (CD-RISC) regarding psychometric properties and components.]. *Z Für Gesundheitspsychologie*. 2015;23(3):112-122. doi:10.1026/0943-8149/a000142

31. Wollny AI, Jacobs I. Validity and reliability of the German versions of the CD-RISC-10 and CD-RISC-2. *Curr Psychol*. 2023;42(5):3437-3448. doi:10.1007/s12144-021-01670-2

32. Development of the World Health Organization WHOQOL-BREF quality of life assessment. The WHOQOL Group. *Psychol Med*. 1998;28(3):551-558. doi:10.1017/s0033291798006667

33. International Council for Harmonisation of Technical Requirements for Pharmaceuticals for Human Use (ICH). ICH E9 (R1) Addendum on Estimands and Sensitivity Analysis in Clinical Trials to the Guideline on Statistical Principles for Clinical Trials. Published online 2019. Accessed October 10, 2024. https://www.ich.org/page/efficacy-guidelines

34. Schramm E, Thiel N. Interpersonelle Psychotherapie bei Arbeitsstress in der Gruppe (IPT-Work).

35. Meister R, Lanio J, Fangmeier T, et al. Adverse events during a disorder-specific psychotherapy compared to a nonspecific psychotherapy in patients with chronic depression. *J Clin Psychol*. 2020;76(1):7-19. doi:10.1002/jclp.22869

36. Rush AJ, Trivedi MH, Ibrahim HM, et al. The 16-Item quick inventory of depressive symptomatology (QIDS), clinician rating (QIDS-C), and self-report (QIDS-SR): a psychometric evaluation in patients with chronic major depression. *Biol Psychiatry*. 2003;54(5):573-583. doi:10.1016/S0006-3223(02)01866-8

37. S3-Leitlinie Nationale VersorgungsLeitlinie Unipolare Depression. Accessed October 1, 2024. https://register.awmf.org/de/leitlinien/detail/nvl-005

38. Kerry SM, Bland JM. Statistics notes: The intracluster correlation coefficient in cluster randomisation. *BMJ*. 1998;316(7142):1455-1460. doi:10.1136/bmj.316.7142.1455

39. Rubin DB. *Multiple Imputation for Nonresponse in Surveys*. 1st ed. Wiley; 1987. doi:10.1002/9780470316696

40. Two-sided confidence intervals for the single proportion: comparison of seven methods - Newcombe - 1998 - Statistics in Medicine - Wiley Online Library. Accessed October 1, 2024. https://onlinelibrary.wiley.com/doi/10.1002/(SICI)1097-0258(19980430)17:8%3C857::AID-SIM777%3E3.0.CO;2-E
